# Dropping in on lipid droplets: insights into cellular stress and cancer

**DOI:** 10.1042/BSR20180764

**Published:** 2018-09-19

**Authors:** Peter Shyu, Xing Fah Alex Wong, Karen Crasta, Guillaume Thibault

**Affiliations:** 1 School of Biological Sciences, Nanyang Technological University, Singapore, 637551; 2 Lee Kong Chian School of Medicine, Nanyang Technological University, Singapore, 308232; 3 Institute of Molecular and Cell Biology, Agency for Science, Technology, and Research, Singapore, 138673; 4 Department of Medicine, Imperial College London, London SW7 2AZ, U.K.

**Keywords:** cell stress, cancer progression, chemoresistance, lipid droplets

## Abstract

Lipid droplets (LD) have increasingly become a major topic of research in recent years following its establishment as a highly dynamic organelle. Contrary to the initial view of LDs being passive cytoplasmic structures for lipid storage, studies have provided support on how they act in concert with different organelles to exert functions in various cellular processes. Although lipid dysregulation resulting from aberrant LD homeostasis has been well characterised, how this translates and contributes to cancer progression is poorly understood. This review summarises the different paradigms on how LDs function in the regulation of cellular stress as a contributing factor to cancer progression. Mechanisms employed by a broad range of cancer cell types in differentially utilising LDs for tumourigenesis will also be highlighted. Finally, we discuss the potential of targeting LDs in the context of cancer therapeutics.

## Introduction

Lipids constitute a class of biomolecules with species as diverse as they are functionally versatile. Not only are these molecules integral to cellular structure, but they are also vital in key biological processes such as energy production, cell signalling and gene regulation among many others [1]. However, despite their physiological importance and implication in many disease states, only in recent years has the study of lipid homeostasis and dynamics found its renaissance.

**Figure 1:**
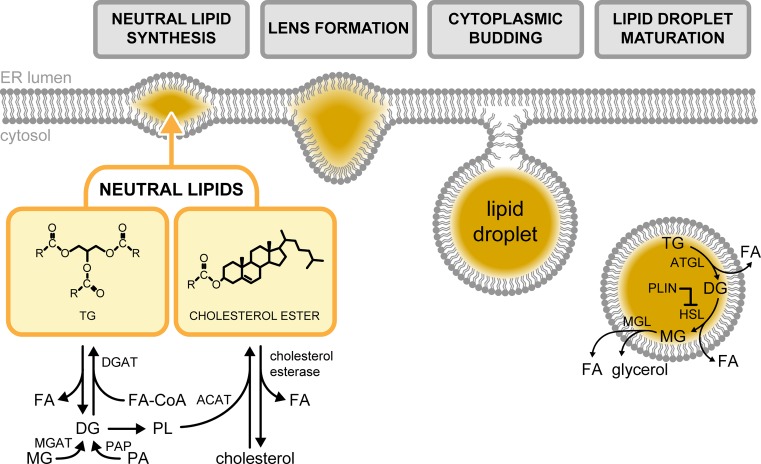
Lipid droplet biogenesis The neutral lipids, TG and cholesterol ester, are synthesised by ER membrane proteins. These lipid molecules then coalesce to form the core of a lens-shaped structure that protrudes towards the cytoplasm. The nascent LD buds off from the ER membrane to form the mature cytoplasmic organelle. Abbreviations: ATGL, adipose triglyceride lipase; DG, diacylglycerol; DGAT, diacylglycerol acyl-transferase; FA-CoA, fatty acyl-CoA; HSL, hormone-sensitive lipase; MG, monoacylglycerol; MGAT, monoacylglycerol acyl-transferase; MGL, monoacylglycerol lipase; PA, phosphatidic acid; PAP, phosphatidic acid phosphatase; PL, phospholipids.

Lipid droplets (LD) are intracellular storage organelles of neutral lipids (NL). Although prominently found in adipose tissue, LDs can exist virtually in all cell types and tissues [2–4]. While these cytoplasmic structures have long been thought to be inert reservoirs for surplus lipids, it became clear more than half a century ago that LDs are dynamic and are exploited by cancer cells [5]. Moreover, only in recent years has the interest on the indispensable role of LDs in cancer progression and therapeutics emerged. Based on their inherent structural properties, cellular lipids may broadly be classified as membrane or non-membrane lipids. The former is primarily composed of amphipathic glycerophospholipids, sphingolipids and sterols, while fatty acids (FAs) and their derivatives are categorised under the latter. Maintenance of cellular lipid levels is governed by the constant flux between its various forms as mediated by several biosynthetic enzymes. However, a distinct class known as NLs forms a non-reactive pool of lipid molecules that serve both as a reserve for intermediates or repository for the surplus of lipids [1,6]. The defining characteristic of LDs as an organelle is its core of NLs. Due to their highly non-polar nature, NLs such as triacylglycerols (TG) and steryl esters (SE) are insoluble in the polar cytoplasmic environment and are not readily incorporated into amphipathic cellular membranes (Figure 1). They instead coalesce and form the core of LDs subsequently after synthesis within the double-membrane leaflet of the endoplasmic reticulum (ER) [7–10]. Thus, the structure of these dynamic cellular organelles is relatively simple, wherein the NL core is surrounded by an ER-derived phospholipid monolayer, with phosphatidylcholine as the major phospholipid component followed by phosphatidylinositol and phosphatidylethanolamine [11]. Moreover, the single phospholipid coat of LDs selectively houses a subset of proteins. Most of these are involved in lipid metabolism such as sterol biosynthetic enzymes and lipases [12–15], while other non-conventional proteins have also been identified [16].

Over the last decade, scientific advancements have conversely provided evidence on the dynamic nature of LDs. An increasing body of study has identified the close physical and functional apposition of LDs to other organelles across different model organisms, as *in vitro* and *in vivo* studies have established the connection between LDs and cellular compartments such as the mitochondria [17–19], the proteasome and the autophagic machinery [20,21]. The association between LDs and various cellular organelles lends support to the role of LDs in a broad range of cellular processes and protein quality control should LD homeostasis be dysregulated [22–25]. Although the appreciation for LDs have grown significantly, apart from studies detailing proteins that influence LD formation [7,26–28], definitive insight on the fundamental events that govern its biogenesis and functioning remains largely enigmatic to this day. Furthermore, these mechanistic studies have been conducted primarily in the unicellular model organism, *Saccharomyces cerevisiae*. Although this may be due to the robust genetic tools available for biochemical manipulation in the budding yeast, it also alludes to the increasing complexity of LD biogenesis in higher eukaryotes. Interestingly, while mutations in proteins associated with LD formation in *S. cerevisiae* yield modest phenotypes under physiological conditions, gross and more severe defects were associated in higher organisms with the corresponding genetic background. For example, deletion of seipin (*SEI1*) in yeast caused a delay in *de novo* LD formation with aberrant morphology, but otherwise yielded minimal effect on cell growth [27]. However, human seipin, also known as the Berardinelli-Seip congenital lipodystrophy 2 gene (*BSCL2*), was found to not only be critical for adipocyte differentiation and lipogenesis induction using *in vitro* cell cultures [29], but is also linked to a more severe form of congenital general lipodystrophy characterised by insulin resistance, hepatic steatosis and extreme reduction in both metabolically active and mechanical adipose tissue in patient studies [30]. Similarly, loss of the fat storage-inducing 2 (*FIT2*) yeast gene homologues impaired LD maturation without affecting general cellular fitness, but a corresponding loss of homologues resulted in early death in both the nematode *Caenorhabditis elegans* and mouse models [28,31]. All these lend support to the role of LDs in both organismal development and metabolic disease predisposition.

As mentioned earlier, LDs have been strongly implicated in cancer progression. However, the current inseparability of LD formation from the synthesis and turnover of its constituent NLs and phospholipids remains to be a caveat that needs to be addressed to ascertain the contribution of LD to tumourigenesis as a fully functional organelle. To date, most studies only focused on the partial functions of the highly dynamic and complex nature of LDs. This review presents different models on the direct and stress-regulatory roles of LDs in cancer cells based on our current understanding of LD biology.

## Cellular stress en route to tumourigenesis: the LD connection

The altered metabolic activity in highly proliferative cancer cells warrants the need for understanding adaptive remodelling of key players in bioenergetics. LDs are among the most integral organelles in this process, and are increasingly identified in various cancer cell types [32]. Furthermore, cancer cells are characterised by elevated cellular stress factors and the activation of their corresponding adaptive response pathways. Concomitantly, the occurrence of LDs is increased under the same stress conditions [33–36]. This then presents the question of whether LD formation potentially aids in stress adaptive responses or contributes to consequences of disrupted cellular homeostasis. Furthermore, how LDs impact stress response regulation in cancer cells is less understood.

### Unfolded protein response in cancer

**Figure 2:**
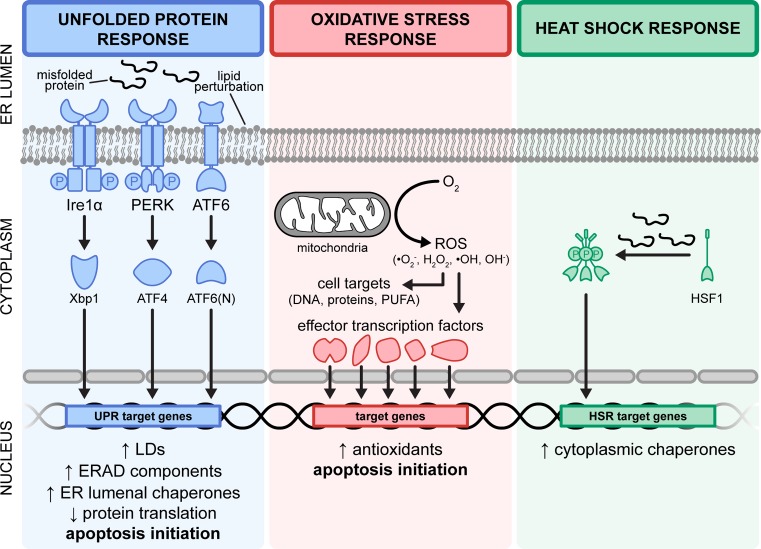
Fundamental activation mechanism of major cellular stress responses (**Left panel**) The UPR is activated by ER stress conditions (i.e. ER membrane perturbation and aberrant protein folding within the ER lumen). These stressors are affected by three distinct axes, namely Ire1α, PERK and ATF6. The cognate transcription factors, Xbp1, ATF4 and ATF6(N), then translocate into the nucleus to modulate gene expression including those of ER luminal chaperones and ERAD components. UPR activation concurrently attenuates global protein translation and induces LD formation to restore homeostasis. (**Middle panel**) Oxidative stressors in the form of reactive oxygen species are generated through energy metabolism. Accumulation of ROS potentially induces a cascade of events that damages DNA, proteins and long-chain PUFAs. This cellular threat is mitigated by the up-regulation of antioxidants that neutralise ROS. The failure to restore homeostasis under conditions of ER and oxidative stress ultimately results in apoptosis. (**Right panel**) In contrast with the UPR, the presence of misfolded proteins in the cytoplasm as well as elevated temperatures activates the heat shock response (HSR). Under these conditions, the monomeric HSF1 sensor forms the homotrimeric HSF1 transcription factor that translocates into the nucleus to increase expression of cytoplasmic protein chaperones to subsequently aid in the refolding and processing of misfolded client substrates. Abbreviations: ERAD, ER-associated degradation; PUFA: polysaturated fatty acid; ROS, reactive oxygen species.

The unfolded protein response (UPR) is a stress response pathway canonically activated from the accumulation of misfolded proteins within the ER lumen, but has since been shown to be similarly triggered upon exposure to exogenous free fatty acids (FFAs) and phospholipid perturbation [37–39], especially that of the ER membrane. This adaptive response pathway aims to restore ER homeostasis by modulating the expression of downstream target genes, and alternatively drives pro-apoptotic pathways should the stress condition remain unresolved. In metazoans, the UPR is mediated by signalling cascade events affected by three distinct ER transmembrane proteins: inositol-requiring enzyme 1α (Ire1α), PRKR-like endoplasmic reticulum kinase (PERK) and activating transcription factor 6 (ATF6), the most evolutionarily conserved and well-studied from yeast to humans being the Ire1 axis (Figure 2). Although there are variations in the intensity of UPR activation as well as differential regulation of downstream target genes dependent on the cause of stress [40–43], both protein- and lipid-induced UPR activation similarly result in increased lipogenic markers and subsequently LD formation [33,34,44], and mutants incapable of LD formation up-regulate the UPR, thus strongly indicative of a role for LDs under the UPR programme. However, the dispensability of NL synthesis for viability under ER stress conditions [33] suggests that the constituent LD core may not be the sole contributor to the homeostatic response and that LDs have another function in protein-induced ER stress.

Consistent with this, the ablation of LD formation by genetic or pharmacological disruption of NL synthesis is dispensable for ER-associated protein degradation (ERAD) function in both yeast and mammalian cell models [45,46]. Proteins failing to fold correctly in the ER are retained and targeted towards ERAD, the terminal phase of ER quality control (Figure 2) [44]. When the pathway is saturated, it leads to the activation of the UPR from an accumulation of misfolded proteins. The ERAD-associated protein ancient ubiquitous protein 1 that mediates substrate ubiquitination has both been found to localise to the LD membrane and to regulate LD abundance [47]. A potential function of LDs in response to protein stress is by serving as temporary depot for proteins destined for degradation [48]. In line with this, HMG-CoA, the rate-limiting enzyme in cholesterol biosynthesis, is shuttled onto LDs as a part of its endogenous degradation mechanism [49]. Similarly, ubiquitinated apoB localises to LDs at sites in contact with the proteasome, and is further supported by the co-fractionation of LDs with ubiquitinated apoB and 20S proteasomal subunits [20]. From these, it may be hypothesised that apart from its NL core, the LD phospholipid membrane may serve a proteostatic function, specifically by housing client proteins that are marked for degradation.

Most recently, increased transcripts of the critical ERAD-associated ubiquitin ligase derlin 3 (*DERL3*) have been reported in metastatic patient-derived breast cancer samples, correlating with poor disease prognosis [50]. Inhibition of *DERL3* expression substantially abrogated proliferation and invasiveness in breast cancer cell lines, while pharmacological impairment of two other steps in ERAD by the anti-cancer agents Eeyarestatin I or Bortezomib-promoted cell death in the JeKo-1 mantle cell lymphoma cell line [50,51]. From these, it could be argued that precluding the clearance of aberrantly misfolded proteins induces apoptosis by increasing the proteotoxic load beyond manageable levels. Consequently, the up-regulation of the proapoptotic factor ATF4 under the PERK-axis of the UPR, upon blocking ERAD [51], provided a close link between the UPR and cancer progression but not a definitive rationale for the increase in LDs that correlate with UPR activation.

Blocking proteasomal degradation by MG132 causes ubiquitinated HMG-CoA to associate with LDs [49]. Despite the non-essentiality of LDs in ERAD, preventing LD formation by the fatty acid synthesis inhibitor triacsin C has been shown to cause increased apoptosis along with the up-regulation of the proapoptotic IRE1- and PERK-axes of the UPR [46]. Taken together, LDs may serve as a safety net for the proteotoxic stress, albeit with an unclear mechanism, should the ERAD network be limited and without which the cell is unable to cope with the proteotoxic load resulting in cell death.

Interestingly, the disruption of the circadian clock by genetic mutation of the Clock and Bmal1 transcriptional regulators have been shown to decrease lipolysis and cause the accumulation of TG in white adipocytes [52], in agreement with low mRNA levels of *Bmal1* and the related regulatory genes *Per2, Rev-erbα* and *Cry1* in obese mice models that develop severe hepatosteatosis [53]. These are strongly indicative of the role of circadian clock as a key regulator of lipid homeostasis. Moreover, the circadian clock is tightly coordinated with temporal UPR activation [54], and is therefore capable of modulating the protein quality control effectors of the UPR. In line with this, it has recently been shown that PERK-mediated UPR activation led to temporal suppression of both *Clock* and *Bmal1* and promoted cancer cell survival by attenuating protein synthesis [55], while the overexpression of *Bmal1* sensitised colorectal cancer cells (CRCs) to chemotherapeutics [56]. Perhaps, pathways that increase LD formation additionally orchestrate a general protein homeostatic response (i.e. ERAD or inhibition of protein synthesis) to prevent proteotoxic stress in cancer cells.

Despite the number of studies that suggest of its protective effects against ER stress, the abundance of cytoplasmic LDs appears to be selectively advantageous for cancer cells and is otherwise cytotoxic in normal cells. The incretin mimetic drug exendin-4 has been shown to reduce accumulation of ER stress-induced LDs with a concomitant decrease in UPR markers in cancer and non-cancer cells [57,58]. It exhibited anti-tumour effects [59] but promoted survival of normal cells [60–62]. Therefore, LDs may not confer an all-encompassing protective effect, but instead buffer against a form of cellular aberration that is exclusively present in cancer cells. An increase in lipogenic enzyme markers has been extensively reported in a variety of cancer cells [63–65], and the resultant flux of lipids warrants a heightened capacity to store them in the form of LDs, a possible route to prevent ER stress induction [38]. However, it was recently reported that while ablation of lipogenic factors prevented cancer progression *in vitro*, it conversely increased tumourigenesis in an *in vivo* hepatocarcinoma mouse model [66]. Although this does not dismiss the plausible contribution of LDs in stress-adaptive tumourigenic processes, it suggests of alternative LD functions and similarly provides a strong impetus to investigate the role of LDs under the complex environment of *in vivo* systems.

### Oxidative stress and cancer

Increased oxidative stress has been observed in several disease states [67–70], a condition characterised by elevated levels of reactive oxygen species (ROS). The generation of ROS naturally occurs through the course of cellular metabolism but could lead to detrimental effects upon accumulation beyond physiological levels. To mitigate this threat, antioxidants (e.g. superoxide dismutases; SODs) sequester and convert ROS into innocuous forms which are further neutralised by the cell [71,72]. Introduction of oxidative stressors and the reduction in antioxidant capability of cells similarly resulted in the increased formation of LDs [22,73,74], suggestive of the buffering capacity of LDs under these conditions. Conversely, knockdown of the LD-stabilising protein perilipin-5 (PLIN5) in an ischaemia-reperfusion mouse model not only resulted in decreased LD pool in myocardial tissue, but also impaired SOD levels and elevated ROS along with severely damaged mitochondria [75]. Similarly in clear-cell renal cell carcinoma (RCC), LD-associated perilipin-2 (PLIN2) expression was found to be regulated by ROS-related hypoxia-inducible factor 2α, resulting in a decrease in FA transport from LDs into the mitochondria [76].

Mitochondria are key sites of lipid metabolism, critical for both energetics and conversion of key lipid species. Being the hub for aerobic respiration, it is also a major site of ROS generation (Figure 2) [77,78]. Without the protective effect of antioxidants, this would be deleterious as lipids, especially polyunsaturated fatty acids (PUFAs), are highly susceptible to peroxidation by ROS [79], and the latter consequently generates toxic by-products that further cause cellular dysfunction. The antioxidant capacity of LDs was demonstrated in *Drosophila* glial cells by the modulation of ROS levels and prevention of lipid peroxidation [80]. Accordingly, incorporation of FFA into NL and storage into LD can be a protective mechanism against peroxidative damage.

Paradoxically, high ROS levels are one of the defining characteristics of various cancer types and is opportunistically utilised by cancerous cells to drive its proliferative and metastatic capacities [81,82]. The current model is that highly metabolic cancer cells generate increased levels of ROS in the mitochondria, as well as up-regulate antioxidant levels. Although higher than non-cancerous cells, ROS levels in cancer cells do not drive apoptosis but instead fuel cell growth and survival through various pathways [83]. This may reflect a general increase in oxidative stress tolerance inherent within cancer cells *per se*, or alternatively a specific consequence of their bolstered antioxidant capacity.

In support of the latter, a wide variety of cancer types significantly up-regulate the antioxidants Sod2, glutaredoxin and glutathione peroxidases [84]. Recent studies also suggest that this increase in oxidative stress tolerance could be extended to adjacent cells and the tumour microenvironment. Cancer cells could alter the proteome of neighbouring cells to its advantage, as evidenced by up-regulation of antioxidants among other proliferative factors in normal fibroblasts co-cultured with breast cancer cells [85]. Similarly, extracellular vesicles in the form of exosomes in conditioned media from pancreatic cancer cells have been reported to house and carry transcripts of *SOD2*, and decreased ROS production in cells treated with these exosomes [86]. Curbing this antioxidant advantage by preventing their expression conversely resulted in increased therapeutic susceptibility [87,88]. As reservoirs of cellular lipids, LDs serve not only to store but also to protect their contents from potential peroxidation, thereby providing yet another avenue for oxidative stress resistance in cancer cells. This is in line with what has been reported for breast cancer cells wherein induction of LD biogenesis conferred resistance to and reduced oxidative stress levels [89]. Mechanistically, this was proposed to be due to the protection of PUFAs from peroxidation by sequestering these toxic lipid by-products into LDs.

A strong evidence for the increased antioxidant capacity of cancer cells came with the recent report of heightened hepatocarcinoma aggressiveness coupled with increased resistance to oxidative agents *in vivo* following the ablation of FA synthesis by *ACC1/2* depletion [66]. While devoid of *de novo* FA biosynthesis, liver-specific *ACC1/2* null mice unexpectedly increased TG accumulation and heightened hepatosteatosis, which was further linked to the decrease in lipid oxidation pathways [90]. This in turn greatly emphasises that the retention of LDs is advantageous not merely for bioenergetics, but likely for antioxidant properties, as evidenced by the decreased levels of oxidant-induced lipid and DNA damage reported in the same study [66]. As information on protein effectors for LD formation as a biophysical process remains elusive, most studies that characterised the function of LDs have been done with ablation of FA synthesis that elicit broader effects on the metabolic network. Liver-specific *ACC1/2*-ablated mice exhibit a greater shift in the activity of the pentose-phosphate pathway, a bioenergetic pathway that has previously been shown to attenuate oxidative stress-induced cell death [91,92]. Therefore, the increased oxidative stress resistance conferred by *ACC1/2* deletion may not solely be dependent on the persistence of cytoplasmic LDs but could possibly be a secondary effect of metabolic remodelling.

Interestingly, ATF4-mediated UPR activation has been shown to increase protein synthesis and oxidative stress, which ultimately promoted cell death [93]. Similarly, an LD-binding thalidomide analogue exhibited cytotoxicity specifically in cancer cell lines concurrently with the induction of both oxidative and ER stress [94]. It would be important to determine if the LD localisation of this drug affects the biosynthesis or stability of LDs, as this would give invaluable information on how LDs participate in its mechanism of action and more importantly, on how LDs are relevant in the modulation of ER and oxidative stress responses in a cancer-specific manner.

### Heat shock response and cancer

Perhaps less appreciated among stress response activation pathways in cancer cells is the heat shock response (HSR). Originally identified to be triggered by heat stress, the HSR is also activated under conditions of cytoplasmic-misfolded protein accumulation [95,96]. In brief, recognition of the stress stimulus activates the master transcriptional regulator of the HSR, heat shock factor 1 (HSF1), which then up-regulates the expression of proteins to promote protein folding and turnover (Figure 2) [97]. These factors include molecular chaperones and their cognate partners termed as heat shock proteins (HSP) that aid in the maturation, folding and refolding of client proteins.

These elements in the HSR signalling pathway have also been reported to have distinct contributions to cancer progression. The transcriptional regulator HSF1 has been reported to not only be critical to sustain cancer proliferation [98], but also up-regulate a subset of genes exclusively in patient-derived malignant cell specimens that are distinct from the canonical HSR targets. This signature gene expression profile has been associated with colon, lung and breast cancer patients with poor prognosis and survivability [99]. Furthermore, cancer cells can up-regulate HSF1 activity in non-cancer cells within the tumour microenvironment to promote the expression of proliferative and metastatic factors [100]. Whether the contribution of HSF1 to cancer progression is restricted to this specialised transcriptional profile remains to be fully understood, as competitive binding of a synthetic compound to known HSF1 targets was sufficient to reduce cell viability [101]. Downstream in the pathway, various forms of Hsp70 and Hsp90 are also up-regulated in hepatocellular carcinoma (HCC) [102], thought to be essential for mitigating proteotoxicity resulting from the highly aberrant genetic profile of cancer cells. To this end, several molecular inhibitors of these HSPs have been developed, which in turn have exhibited anti-cancer properties [103–105].

On a fundamental level, TG synthesis has been reported to be important in coping with mild heat stress in *Schizosaccharomyces pombe* [106], suggesting a general adaptive role for LDs in response to heat shock*.* Furthermore, the expression of the HSR-associated HSP70 chaperone that is integral to protein folding both under basal and heat stress conditions has been found to be lower in diseased individuals [107] and up-regulated upon exposure to heat stress in an *in vitro* adipocyte model [108]. It was then hypothesised that Hsp70 localises to the LD membrane to facilitate denatured proteins for folding, consistent with that of previous studies [48].

In cancer cells, induction of heat stress resulted in membrane remodelling involving key changes in membrane fluidity to maintain cellular viability and integrity under non-physiological temperatures [109]. Among many factors, cholesterol content is a key determinant of membrane fluidity [110–113]. With this, cholesterol esters (CE) within the core of LDs could be accessed by lipases to liberate their constituent cholesterol moiety for subsequent incorporation into membranes. More recently, the metabolic regulator peroxisome proliferator-activated receptor γ coactivator 1α (PGC-1α) has been identified to serve an anti-proliferative function in prostate cancer cells [114] and repress HSF1 transcription, thereby preventing the otherwise up-regulation of HSR target genes in hepatocellular cancer cells [115]. Interestingly, PLIN5 on the surface of LDs form an active transcriptional complex with PGC-1α upon lipolytic induction [116]. It could therefore be hypothesised that PLIN5 may activate PGC-1α activity to repress HSF1 function. However, with LD accumulation in cancer cells, PLIN5 remains sequestered on the LD membrane, thereby allowing the unrestrained HSR to exert its cancer-promoting functions.

The direct association between the HSR and cancer has long been established with early reports of increased tumour chemoresistance following prolonged or pre-administration of cells to non-lethal heat stress [117,118]. For instance, the prominent downstream Hsp70 effector of the HSR was reported to drive lipogenic pathways in HepG2 cells and increase intracellular LDs [119]. The phospholipase A2 enzyme localises to the surface of LDs from which it catalyses the release of arachidonic acid [120], a strong driver of HSR activation [121]. Arachidonic acid release in cancer cells was also observed to increase from phospholipase activity following exposure to high temperatures [122]. The induction of Hsp70 and the phospholipase-mediated release of arachidonic acid from LDs may then constitute a positive feedback mechanism to increase cancer cell survival under chemotherapeutic stress, as disruption of either phospholipase A2 or Hsp70 improved drug efficacy [123,124].

Undoubtedly, LDs not only exert various functions in cellular stress response pathways, but in turn are also intricately regulated by the activation of the latter. Adding to their complex regulation, LDs interact and communicate with other organelles involving key biological processes. Therefore, it is not surprising that LDs have emerged as critical factors in disease pathogenesis in recent years. However, one of the greatest obstacles to reach a definitive conclusion for the contribution of LDs to cellular stress adaptation is the limitation on methods to uncouple LD formation from FA synthesis. Key scientific findings such as investigating the function of LDs as an independent physical organelle and not as a consequence of lipid biosynthesis *per se*, as well as the identification of definitive regulators for its formation are necessary to further advance the field of LD biology. Nevertheless, the functional roles of LDs are becoming a significant focus of cancer research in anticipation to exploit LD dynamics to curb cancer progression.

## Cancer: a LD-driven metabolic disease

### Correlation of LDs and cancer aggressiveness

Tumour development can be characterised by well-defined hallmarks of cancer that include sustenance of proliferative signalling, evasion of growth suppressors, invasion and metastasis, enabling replicative immortality, angiogenesis and resisting cell death among others [125]. LDs may potentially influence these processes, thus contributing to tumour development and aggressiveness, with potential for LD accumulation as an additional hallmark of cancer. This can be illustrated by reports studying breast cancer cell lines [126,127]. Here, LD abundance was shown to correlate with degree of aggressiveness from the non-malignant MCF10A cells to the highly malignant MDA-MB-231 cells, while MCF7 cells were found to be intermediately aggressive. The higher lipid content in malignant breast cancer cells may arise from the increased rate of FA and phospholipid synthesis, which were found to be essential to drive proliferation in unfavourable harsh environments [128–130]. Furthermore, it has been shown that to prevent nutrient stress and promote proliferation, breast cancer cells import FFAs to either generate energy through β-oxidation or subsequently store them into LDs when present in excess [89]. In another report, the migratory capability of the malignant cell line MDA-MB-231 was also shown to correlate with lipid accumulation [131]. Additionally, high levels of LDs in circulating tumour cells have been associated with cell invasiveness [132]. The CE constituent of LDs was also shown to be associated with human prostate cancer invasiveness and metastasis, while inhibition of CE ester synthesis and storage reduced cancer aggressiveness [133]. Reports have shown cholesterol biogenesis to mostly occur in hepatocytes and adipose tissue [134,135]. Since regulators of cholesterol metabolism, specifically the sterol regulatory element binding proteins (SREBP-1 and SREBP-2) and Δ24-sterol reductase, are present at low levels in renal cells, cholesterol biogenesis is thought to be minimal [136]. However, SREBPs have been reported to be up-regulated in RCC. The *TRC8* gene which encodes a multimembrane-spanning ER protein with E3-ubiquitin ligase activity [137,138] is associated with hereditary RCC as well as ovarian dysgerminoma [139,140]. In RCC, TRC8 destabilises SREBPs at the ER membrane resulting in high levels of soluble SREBPs. Membrane-bound SREBPs sense cholesterol at the ER and are cleaved by sequential enzymes at the Golgi apparatus, releasing soluble SREBPs that function as a transcription factor. In addition to increasing cholesterol biogenesis through stearoyl CoA desaturase (*SCD*), SREBPs also regulate fatty acid synthase (*FASN*). Thus, TRC8 contributes to the increase of LDs in RCC and possibly to its aggressiveness as inhibiting LD synthesis supresses malignancy. Similarly, SREBP-1 has been reported to be overexpressed in prostate cancer cells, inducing an increase in LD biogenesis [141,142]. LD formation has been previously associated with cell survival and cell migration in prostate cancers [142]. Interestingly, androgen treatment increased the number of LDs in prostate cancer cells where SREBP-1 might be implicated [143]. Thus, LDs appear to play an active role in enhancing cancer aggressiveness and could be used as a potential biomarker for metastatic and high-grade cancers. Taken together, all these suggest a role for LDs as a promising target for cancer therapy.

### Function of LDs: interplay between tumour microenvironment, bioenergetics and inflammation

**Figure 3:**
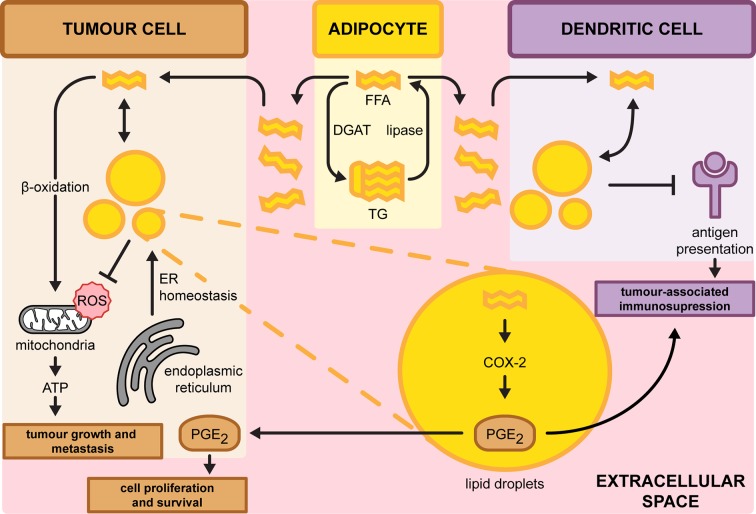
Overview on the role of LDs in regulating cancer progression Tumour cells import FFAs from the extracellular space and adipocyte. Subsequently, these can be stored into LDs to prevent the accumulation of ROS or degraded through β-oxidation to promote tumour growth and metastasis. LD accumulation is also triggered from ER stress to alleviate stress. In dendritic cells (DCs), accumulation of LDs dysregulates antigen presentation of tumour-associated antigens which may inhibit immune response. In parallel, LD-localised prostaglandin E_2_ (PGE_2_) might be secreted causing immunosuppression. In tumour cells, PGE_2_ promotes cell proliferation and cell survival. COX-2, cyclooxygenase-2.

Cancer cells often acquire characteristic changes in their metabolic status [144]. Accordingly, one such change is the enhancement of glycolysis to fuel cancer cell proliferation by supplying energy and precursors for anabolism [145,146]. Lipid metabolism is also altered in highly proliferating cancer cells [147]. The lipogenic genes acetyl-CoA carboxylase (*ACC*), ATP citrate synthase (*ACLY*) and *FASN* have been shown to be up-regulated in various tumours [148–150]. In agreement, increased number of LDs has been reported in many cancer types as excess lipids are stored in LDs to prevent lipotoxicity [5,126,151,152]. Analogous to the role of lipid synthesis in promoting cancer, the breakdown of LD-localised lipids can function as either the primary or alternative source of energy and precursors for phospholipids, the building blocks of biological membranes, in the course of cancer cell proliferation (Figure 3) [152,153]. The connection between LD accumulation and cancer bioenergetics was elegantly demonstrated both *in vitro* and *in vivo* in ovarian cancer cells adjacent to adipocytes [154]. Ovarian cancer cells induce the release of FFAs from TG in adipocytes by intercellular stimulation of adipose triglyceride lipase (ATGL) and hormone-sensitive lipase (HSL) mediated lipolysis (Figure 1). Adipocyte-derived FFAs were also shown to be transported into cancer cells by the fatty acid-binding protein 4 and routed to β-oxidation, contributing to tumour growth and metastasis [155]. Similar findings of FFA transfer from adipocytes were reported in breast and prostate cancer cells [156,157]. Thus, cancer cells opportunistically exploit the surrounding tumour microenvironment to fuel their energetic needs. Consequently, the abundance of lipids stored in adipocytes of overweight patients could be a driver of cancer progression that merits further exploration in future studies.

In addition to adipocytes, the lipid-rich tumour microenvironment could also potentiate defective anti-tumour properties in dendritic cells (DCs) (Figure 3) [158]. DCs with high LD content failed to process or present tumour-associated antigens to stimulate allogeneic T cells in tumour-bearing mice. The accumulation of LDs is a direct consequence of the increased uptake in extracellular lipids. Even in the absence of a lipid-rich microenvironment, a study found that the abnormal build-up of LDs in tumour-associated dendritic DCs could be triggered by the intrinsic ER stress-dependent XBP1 pathway [159]. In both scenarios, suppression of lipid accumulation by 5-(tetradecyloxy)-2-furoic acid (TOFA) restored the functional capacity of DCs and enhanced the activity of the anti-tumour T-cell response [160]. Molecular mechanisms underlying the defective antigen presentation by high LD content remains to be elucidated. Excessive accumulation of LD may interfere with antigen presentation in DCs by sequestering antigenic fragments into LDs, thereby preventing its cell surface display [161]. Protein-LD co-localisation analyses in tumour-associated DCs verified this phenomenon. From these, LD accumulation in cancer cells may modulate the effectiveness of anti-tumour immune responses and therefore pose as a viable target for therapy.

Adenocarcinoma and HCC cell lines were found to contain a significant level of LD-localised cyclooxygenase-2 (COX-2). The enzyme COX-2 is the rate-limiting step in prostaglandin synthesis, which promotes tumour growth as well as coordinates crosstalk with the surrounding stromal cells [162]. LDs then serve as a distinct site for the production of prostaglandin E_2_ (PGE_2_), a potent lipid inflammatory mediator (Figure 3) [163,164]. It is noteworthy that PGE_2_ is the most abundant prostaglandin found in several malignancies including colon, lung and breast tissues [165,166]. PGE_2_ not only promotes tumour growth in a paracrine manner but could also perturb anti-tumour immunity by regulating the activity of the surrounding immune cells in the microenvironment [167,168]. Recent findings reveal that tumour-derived PGE_2_ impaired the viability of natural killer cells and the subsequent recruitment of DCs, resulting in immune evasion [169]. Furthermore, PGE_2_ was also found to suppress natural killer cell activity through the induction of myeloid-derived suppressor cells from the tumour microenvironment [170,171]. These observations suggest that LD accumulation in either tumour or immune cells promotes immunosuppression. Thus, LD could serve as an alternative inflammatory machinery apart from the ER and Golgi apparatus that houses a readily available source of lipids to synthesise vast amounts of pro-inflammatory mediators. LDs could also process lipids acquired from the extracellular environment and efficiently convert them into potent inflammatory products. A better understanding of the role of LDs in regulating the immune response against tumour cells is critical to address cancer-related immunosuppression. Identifying the components of isolated LD and its associated proteins in LD-laden cells by lipidomic and proteomic approaches will unravel integrated lipid biosynthesis pathways responsible for the production of lipid mediators [172,173]. Similar approaches could also be employed to identify other critical LD-associated factors that contribute to cancer aggressiveness in malignant breast cancer cell lines [126,127].

### LD links viral infection to cancer

Hepatitis C virus (HCV) infection is one of the major drivers of HCC [174]. Chronic HCV infection increases inflammation to progressively cause liver damage by stimulating fibrosis and cirrhosis, contributing to HCC development. HCV, through its core protein, dysregulates lipid metabolism, resulting in the development of fatty liver [175]. Although it remains unclear if HCV is involved in tumour initiation or the subsequent inflammatory response, LDs have been shown to play a crucial role in the generation of HCV in infected cells [176]. The HCV core protein promotes the localisation of HCV non-structural proteins from the ER to LDs [176], suggesting that LDs promote the production of the infectious virus particle. HCV triggers the accumulation of LDs which function as a recruitment site for virus assembly, and failure to associate the essential viral proteins and RNAs with LDs impaired the production of the infectious virus [176,177]. The role of LDs in promoting HCV-induced carcinogenesis is also supported by the up-regulation of the PLIN2 biomarker that correlates with proliferation rate during early tumourigenesis [178]. Furthermore, the disruption of LD biogenesis by genetically attenuating the TG-synthesis enzyme diacylglycerol acyltransferase-1 severely reduced viral replication [179]. Collectively, these suggest that oncovirus-induced LDs are indispensable in the development of HCC.

Interaction between viruses and LDs as well as LD formation are not unique to HCV. Dengue virus, part of the *Flaviviridae* single-stranded positive-sense RNA virus family along with HCV, associate with LDs for assembly and RNA replication [180–183]. Viruses of other families can also exploit LDs for their replication. Rotavirus and orthoreovirus interact with LDs as a part of their infection strategy [184,185], while Hepatitis B virus (HBV) X protein expression has been reported to promote LD accumulation [186–188]. Interestingly, HBV was found to induce the up-regulation of transcription factor SREBPs, FASN, as well as FA storage and glucose metabolism regulator PPARγ, all of which contribute to lipid biogenesis and storage. Thus, the role of various viruses in promoting tumourigenesis through the exploitation of LDs could be further studied and exploited therapeutically, especially for chronic infections.

## LDs in chemotherapy

### LDs in cancer patients with cachexia

Cachexia is a complex metabolic syndrome characterised by severe body wasting from the decline in muscle mass and adipose tissue during cancer progression [189]. A hallmark of cachexia is the loss of skeletal muscle and lean tissue together with adipose tissue which consequently leads to physical weakness in patients [190]. Chemotherapy is frequently the underlying cause of cachexia in cancer patients [191]. It was recently proposed that chemotherapy-induced cachexia is driven by oxidative stress-associated MAPK activation in mitochondria of muscle tissues, as in the case of Folfox or Folfiri drug treatments [192]. Elevated levels of plasma FFAs, TG and cholesterol correlate with muscle and adipose tissue wasting in cancer patients with cachexia [193]. Moreover, expression levels of LD-associated proteins are altered in cancer-associated cachexia resulting in changes in adipocyte morphology. This is further supported by the increase in ATGL-dependent lipolysis found in white adipose tissue (WAT) during cancer cachexia [194]. WAT characteristically stores a single oversized LD for energy storage that occupies the bulk of the cytoplasm, while brown adipose tissue (BAT) contains small LDs and an unusually high abundance of mitochondria consequently releasing heat through FA β-oxidation [195]. Increased lipolysis in WAT is also modulated by HSL [193,196]. Although lipid breakdown is the major determinant of cancer cachexia, impaired lipogenic and lipid storage pathways have been closely associated with adipose tissue atrophy [197]. Furthermore, loss of WAT can be due to its gradual conversion to BAT-like cells also known as beige adipocytes [198]. The increase in beige adipocytes may exacerbate the metabolic dysfunction by enhancing lipid mobilisation and driving high energy expenditure in cancer cachexia [189]. The dramatic loss of WAT and its enhanced lipolysis precede the onset of skeletal muscle atrophy and result in the overall depletion of LDs in adipocyte tissue [190]. In contrast, increase in the number and size of intramyocellular LDs had been observed throughout cachexia progression, specifically upon weight loss, in cancer patients [199]. Although the mechanism leading to LD accumulation in muscle cells in cachectic patients is unclear, the dysregulated storage and catabolism of lipids may be the primary underlying cause [189].

### LDs contribution to cell death and therapy-induced senescence

In addition to its role in mediating cancer cell development and proliferation, accumulation of LDs has been widely reported to correlate with chemotherapeutic treatments. For example, LDs have been shown to be a consequence of metabolic changes during etoposide-induced apoptosis, where the inhibition of FA β-oxidation in defective mitochondria re-directs FFAs towards lipid storage (Figure 3) [200]. Indeed, inhibition of β-oxidation or oxidative phosphorylation is sufficient to drive LD formation in cancer cells [201]. Treatment of N-myc proto-oncogene protein-amplified neuroblastoma cells with 10058-F4, an inhibitor of c-MYC/Max protein, a member of the basic helix–loop–helix leucine zipper family of transcription factors, triggers the accumulation of intracellular LDs as an outcome of mitochondrial dysfunction. The chemotherapeutic drugs doxorubicin and 5-fluorouracil (5-FU) in human colon carcinoma cells induce the accumulation of LDs as a result of up-regulated TG biosynthesis [192,202]. Hence, this points to a potential role for LDs as part of a general stress response to different classes of chemotherapeutic treatments. Increased LD formation during chemotherapy may also be a consequence of non-apoptotic cell death (NCD) as ferroptosis-induced NCD perturbed FA metabolism [203,204]. Lipid biosynthesis stimulated the accumulation of FA and initiated NCD in fibrosarcoma HT1080 cell lines [205]. Suppression of either LD or FA accumulation by TOFA reduced lipid dysregulation-associated cell death [206]. Elucidating how LDs assist in mediating cell death, and isolating factors that directly contribute to LD accumulation could be the focus of future studies.

Conventional anticancer treatment in tumour cells may induce a stable cell cycle arrest, a cytostatic phenotype known as ‘therapy-induced senescence’ (TIS). Irreparable DNA damage, chemotherapeutics and ionising radiation all contribute to TIS. Thus, TIS cells have emerged as an exploitable side effect of chemotherapy to prevent cancer progression while reducing toxicity [207]. Recently, autophagy was suggested to be a mechanism contributing to anti-mitotic drug TIS [208]. Although the mechanism was not elucidated, one could envision that lipid metabolism may play a role in promoting autophagy-dependent TIS. Indeed, TIS cells were recently shown to accumulate LDs in contrast with their proliferative counterpart [209]. Here, the accumulation of LDs in TIS from enhanced lipid import was found to contribute to the onset and maintenance of senescence. Excess lipids that could not be stored in overloaded LDs may promote the generation of lipotoxic diacylglycerol and ceramides that potentially trigger stress-induced cellular senescence [210]. This is further supported by the notion that TG or ceramide alone is sufficient to induce senescence in cancer cells. Therefore, accumulation of LDs may be exploited as a means to identify TIS and potentially promote clinical remission through senescence induction.

### LDs contribution to chemoresistance

In contrast with the role of LDs in contributing to cell death, increased LD formation in certain cancer cells correlate with chemotherapy resistance. Chemoresistance is a persistent problem in treating tumour invasion along with the evasion of drug-induced tumour growth inhibition. Resistance varies tremendously among different cancer types, but lipid metabolism can be a common underlying contributor. As such, in oncogene-inactivated residual breast cancer cells, there is a metabolic shift that is associated with the accumulation of LDs [211]. Oncogene ablation-resistant pancreatic cancer cells were shown to accumulate LDs to maintain energetic balance [212]. Recently, LD accumulation in 5-FU and oxaliplatin-treated CRC was also shown to drive cell death resistance by perturbing the caspase activation cascade and ER stress response [213]. Both drugs induce apoptosis by activating the caspase-12 pathway through ER stress. LD accumulation in chemotherapy-induced CRC is dependent on the expression of lysophosphatidylcholine acyltransferase 2 (LPCAT2), an LD-localised enzyme essential for phosphatidylcholine synthesis. The high expression of LPCAT2 is sufficient to prevent the chemotherapy-induced ER stress, underlining the role of LD accumulation in reducing ER stress [214]. More remarkably, it was also reported that LD accumulation correlates with a reduction in immunogenic cell death and CD8^+^ T cell infiltration in mouse xenograft and metastatic tumours of CRC patients, further reinforcing the involvement of LDs in promoting an immunosuppressive tumour environment. The present study reaffirmed the multifaceted role of LDs in mediating chemoresistance by concurrently alleviating chemotherapeutic-induced stress and regulating the tumour microenvironment.

The contribution of LDs to chemoresistance could arise from *de novo* formation following treatment with chemotherapeutic agents or from LDs inherently present in cancer cells. An example of the latter is shown in androgen-sensitive prostate cancer cells that rely on LD degradation and lipolysis for survival during androgen deprivation therapy [215]. Enhanced lipolysis of LD may provide fatty acids for energy production through fatty acid β-oxidation or supply lipid intermediates, particularly CE to promote prostate cancer cell survival [216]. Put into perspective, LD formation is not merely a consequence of the apoptotic cell death response but a hallmark of cell resistance contributing to chemoresistance or disease relapse.

## Conclusion and perspectives

Lipids are essential members of the cellular machinery to regulate proliferation, migration and survival of cancer cells. As part of the equation, LDs from cancer or neighbouring cells play a role in regulating as well as dysregulating some of these processes necessary for cancer proliferation. In cancer cells, the UPR, HSR and oxidative stress response are exploited for survival, resulting in elevated LDs. The field is still in its infancy, and it is imperative that recent promising leads be further explored. Importantly, our current knowledge of how cancer cells exploit LD for critical growth for the development of new therapeutic treatments is still limited and requires in-depth studies. Thus far, no direct inhibitors of LD biogenesis have been developed as anti-proliferative targets.

Indirectly connected to LD regulation, several lipid biogenesis inhibitors are currently under preclinical development including those that specifically target FA regulation [147,217]. Among these are inhibitors of biosynthetic enzymes for the acetyl-CoA precursor (ACLY) [218], FA synthesis (FASN) [219], FA elongation [220], FA desaturation (SCD1-5) [221] and FA β-oxidation (CPT1) [222–224], which could potentially be used to deter cancer cells that exploit LDs for growth. These inhibitors will need to attenuate tumourigenesis without dysregulating body weight. However, as mentioned earlier, no specific LD inhibitors have been developed to date and should be an avenue of exploration. Inhibition of LD formation results in toxic accumulation of FFAs promoting cell death [225,226]. To inhibit LD formation, genetic attenuation or small molecules targeting FIT1, FIT2 or PLIN should be considered. As LD biogenesis is still poorly understood, the urgent need to identify novel protein factors will be critical for the development of new inhibitors to prevent tumour growth. A combination with LD inhibitors might enhance the efficacies of existing treatments. Drugs that specifically target LD formation hold greater therapeutic potential compared with general lipid biosynthesis inhibitors as the accumulation of LDs confer survival advantages in cancer cells. Specifically reducing LD accumulation in tumour might reduce drug toxicity on non-malignant cells. In contrast, drugs targeting precursors of FAs, phospholipids, cholesterol, sphingolipids or ceramides might lead to undesirable side effects as these lipids are involved in a large variety of cellular processes. All in all, specific inhibitors against LD biogenesis or the utility of LD as biomarkers in certain cancers could open up a new class of therapeutics or prognostication in cancers.

## Abbreviations

5-FU: 5-fluorouracil
ACC: acetyl-CoA carboxylase
ACLY: ATP citrate synthase
ATF6: activating transcription factor 6
ATGL: adipose triglyceride lipase
BAT: brown adipose tissue
CE: cholesterol ester
COX-2: cyclooxygenase-2
CRC: colorectal cancer
DC: dendritic cell
ER: endoplasmic reticulum
ERAD: ER-associated protein degradation
FA: fatty acid
FFA: free fatty acid
FASN: fatty acid synthase
HBV: hepatitis B virus
HCV: hepatitis C virus
HSL: hormone-sensitive lipase
HSR: heat shock response
Ire1α: inositol-requiring enzyme 1α
LD: lipid droplet
LPCAT2: lysophosphatidylcholine acyltransferase 2
MAPK: mitogen-activated protein kinase
NCD: non-apoptotic cell death
NL: neutral lipid
PERK: PRKR-like endoplasmic reticulum kinase
PGC-1α: peroxisome proliferator-activated receptor γ coactivator 1α
PGE_2_: prostaglandin E_2_
PUFA: polyunsaturated fatty acid
RCC: renal cell carcinoma
ROS: reactive oxygen species
SCD: stearoyl CoA desaturase
SE: steryl ester
SREBP: sterol regulatory element biding protein
SOD: superoxide dismutase
TIS: therapy-induced senescence
TG: triacylglycerol
TOFA: 5-(tetradecyloxy)-2-furoic acid
UPR: unfolded protein response
WAT: white adipose tissue

## References

[1] van Meer G., Voelker D.R. and Feigenson G.W. (2008) Membrane lipids: where they are and how they behave. Nat. Rev. Mol. Cell Biol. 9, 112–124 10.1038/nrm2330 18216768 PMC2642958

[2] Lynch K.M. Jr and Scott W.W. (1951) Lipid distribution in the Sertoli cell and Leydig cell of the rat testis as related to experimental alterations of the pituitary-gonad system. Endocrinology 49, 8–14 10.1210/endo-49-1-8 14872818

[3] Montagna W., Chase H.B. and Hamilton J.B. (1951) The distribution of glycogen and lipids in human skin. J. Invest. Dermatol. 17, 147–157 10.1038/jid.1951.75 14880730

[4] Fawcett D.W. (1955) Observations on the cytology and electron microscopy of hepatic cells. J. Natl. Cancer Inst. 15, 1475–1503 13243087

[5] Apffel C.A. and Baker J.R. (1964) Lipid droplets in the cytoplasm of malignant cells. Cancer 17, 176–184 10.1002/1097-0142(196402)17:2%3c176::AID-CNCR2820170207%3e3.0.CO;2-2 14123678

[6] Zweytick D., Leitner E., Kohlwein S.D., Yu C., Rothblatt J. and Daum G. (2000) Contribution of Are1p and Are2p to steryl ester synthesis in the yeast Saccharomyces cerevisiae. Eur. J. Biochem. 267, 1075–1082 10.1046/j.1432-1327.2000.01103.x 10672016

[7] Szymanski K.M., Binns D., Bartz R., Grishin N.V., Li W.P., Agarwal A.K. (2007) The lipodystrophy protein seipin is found at endoplasmic reticulum lipid droplet junctions and is important for droplet morphology. Proc. Natl. Acad. Sci. U.S.A. 104, 20890–20895 10.1073/pnas.0704154104 18093937 PMC2409237

[8] Jacquier N., Choudhary V., Mari M., Toulmay A., Reggiori F. and Schneiter R. (2011) Lipid droplets are functionally connected to the endoplasmic reticulum in Saccharomyces cerevisiae. J. Cell Sci. 124, 2424–2437 10.1242/jcs.076836 21693588

[9] Soni K.G., Mardones G.A., Sougrat R., Smirnova E., Jackson C.L. and Bonifacino J.S. (2009) Coatomer-dependent protein delivery to lipid droplets. J. Cell Sci. 122, 1834–1841 10.1242/jcs.045849 19461073 PMC2684835

[10] Ohsaki Y., Cheng J., Suzuki M., Fujita A. and Fujimoto T. (2008) Lipid droplets are arrested in the ER membrane by tight binding of lipidated apolipoprotein B-100. J. Cell Sci. 121, 2415–2422 10.1242/jcs.025452 18577578

[11] Tauchi-Sato K., Ozeki S., Houjou T., Taguchi R. and Fujimoto T. (2002) The surface of lipid droplets is a phospholipid monolayer with a unique fatty acid composition. J. Biol. Chem. 277, 44507–44512 10.1074/jbc.M207712200 12221100

[12] Athenstaedt K., Zweytick D., Jandrositz A., Kohlwein S.D. and Daum G. (1999) Identification and characterization of major lipid particle proteins of the yeast Saccharomyces cerevisiae. J. Bacteriol. 181, 6441–6448 10515935 10.1128/jb.181.20.6441-6448.1999 PMC103780

[13] Fujimoto Y., Itabe H., Sakai J., Makita M., Noda J., Mori M. (2004) Identification of major proteins in the lipid droplet-enriched fraction isolated from the human hepatocyte cell line HuH7. Biochim. Biophys. Acta 1644, 47–59 10.1016/j.bbamcr.2003.10.018 14741744

[14] Brasaemle D.L., Dolios G., Shapiro L. and Wang R. (2004) Proteomic analysis of proteins associated with lipid droplets of basal and lipolytically stimulated 3T3-L1 adipocytes. J. Biol. Chem. 279, 46835–46842 10.1074/jbc.M409340200 15337753

[15] Bersuker K. and Olzmann J.A. (2018) In Close Proximity: the lipid droplet proteome and crosstalk with the endoplasmic reticulum. Contact 1, 10.1177/2515256418768996 PMC6083863 30101213

[16] Cho S.Y., Shin E.S., Park P.J., Shin D.W., Chang H.K., Kim D. (2007) Identification of mouse Prp19p as a lipid droplet-associated protein and its possible involvement in the biogenesis of lipid droplets. J. Biol. Chem. 282, 2456–2465 10.1074/jbc.M608042200 17118936

[17] Blanchette-Mackie E.J. and Scow R.O. (1983) Movement of lipolytic products to mitochondria in brown adipose tissue of young rats: an electron microscope study. J. Lipid Res. 24, 229–244 6842081

[18] Pu J., Ha C.W., Zhang S., Jung J.P., Huh W.-K. and Liu P. (2011) Interactomic study on interaction between lipid droplets and mitochondria. Protein Cell 2, 487–496 10.1007/s13238-011-1061-y 21748599 PMC4875178

[19] Wang H., Sreenivasan U., Hu H., Saladino A., Polster B.M., Lund L.M. (2011) Perilipin 5, a lipid droplet-associated protein, provides physical and metabolic linkage to mitochondria. J. Lipid. Res. 52, 2159–2168 10.1194/jlr.M017939 21885430 PMC3220284

[20] Ohsaki Y., Cheng J., Fujita A., Tokumoto T. and Fujimoto T. (2006) Cytoplasmic lipid droplets are sites of convergence of proteasomal and autophagic degradation of apolipoprotein B. Mol. Biol. Cell 17, 2674–2683 10.1091/mbc.e05-07-0659 16597703 PMC1474802

[21] Shibata M., Yoshimura K., Furuya N., Koike M., Ueno T., Komatsu M. (2009) The MAP1-LC3 conjugation system is involved in lipid droplet formation. Biochem. Biophys. Res. Commun. 382, 419–423 10.1016/j.bbrc.2009.03.039 19285958

[22] Nguyen T.B., Louie S.M., Daniele J.R., Tran Q., Dillin A., Zoncu R. (2017) DGAT1-dependent lipid droplet biogenesis protects mitochondrial function during starvation-induced autophagy. Dev. Cell 42, 9e5–21e5 10.1016/j.devcel.2017.06.003 28697336 PMC5553613

[23] Rambold A.S., Cohen S. and Lippincott-Schwartz J. (2015) Fatty acid trafficking in starved cells: regulation by lipid droplet lipolysis, autophagy, and mitochondrial fusion dynamics. Dev. Cell 32, 678–692 10.1016/j.devcel.2015.01.029 25752962 PMC4375018

[24] Tsai T.H., Chen E., Li L., Saha P., Lee H.J., Huang L.S. (2017) The constitutive lipid droplet protein PLIN2 regulates autophagy in liver. Autophagy 13, 1130–1144 10.1080/15548627.2017.1319544 28548876 PMC5529083

[25] Weisshaar N., Welsch H., Guerra-Moreno A. and Hanna J. (2017) Phospholipase Lpl1 links lipid droplet function with quality control protein degradation. Mol. Biol. Cell 28, 716–725 10.1091/mbc.e16-10-0717 28100635 PMC5349779

[26] Adeyo O., Horn P.J., Lee S., Binns D.D., Chandrahas A., Chapman K.D. (2011) The yeast lipin orthologue Pah1p is important for biogenesis of lipid droplets. J. Cell Biol. 192, 1043–1055 10.1083/jcb.201010111 21422231 PMC3063132

[27] Cartwright B.R., Binns D.D., Hilton C.L., Han S., Gao Q. and Goodman J.M. (2015) Seipin performs dissectible functions in promoting lipid droplet biogenesis and regulating droplet morphology. Mol. Biol. Cell 26, 726–739 10.1091/mbc.e14-08-1303 25540432 PMC4325842

[28] Choudhary V., Ojha N., Golden A. and Prinz W.A. (2015) A conserved family of proteins facilitates nascent lipid droplet budding from the ER. J. Cell. Biol. 211, 261–271 10.1083/jcb.201505067 26504167 PMC4621845

[29] Payne V.A., Grimsey N., Tuthill A., Virtue S., Gray S.L., Dalla Nora E. (2008) The human lipodystrophy gene BSCL2/seipin may be essential for normal adipocyte differentiation. Diabetes 57, 2055–2060 10.2337/db08-0184 18458148 PMC2494687

[30] Simha V. and Garg A. (2003) Phenotypic heterogeneity in body fat distribution in patients with congenital generalized lipodystrophy caused by mutations in the AGPAT2 or seipin genes. J. Clin. Endocrinol. Metab. 88, 5433–5437 10.1210/jc.2003-030835 14602785

[31] Goh V.J., Tan J.S., Tan B.C., Seow C., Ong W.Y., Lim Y.C. (2015) Postnatal deletion of fat storage-inducing transmembrane protein 2 (FIT2/FITM2) causes lethal enteropathy. J. Biol. Chem. 290, 25686–25699 10.1074/jbc.M115.676700 26304121 PMC4646211

[32] Tirinato L., Pagliari F., Limongi T., Marini M., Falqui A., Seco J. (2017) An overview of lipid droplets in cancer and cancer stem cells. Stem Cells Int. 2017, 1656053 10.1155/2017/1656053 28883835 PMC5572636

[33] Fei W., Wang H., Fu X., Bielby C. and Yang H. (2009) Conditions of endoplasmic reticulum stress stimulate lipid droplet formation in Saccharomyces cerevisiae. Biochem. J. 424, 61–67 10.1042/BJ20090785 19708857

[34] Lee J.S., Mendez R., Heng H.H., Yang Z.Q. and Zhang K. (2012) Pharmacological ER stress promotes hepatic lipogenesis and lipid droplet formation. Am. J. Transl. Res. 4, 102–113 22347525 PMC3276380

[35] Gubern A., Barceló-Torns M., Casas J., Barneda D., Masgrau R., Picatoste F. (2009) Lipid droplet biogenesis induced by stress involves triacylglycerol synthesis that depends on group VIA phospholipase A2. J. Biol. Chem. 284, 5697–5708 10.1074/jbc.M806173200 19117952

[36] Yamamoto K., Takahara K., Oyadomari S., Okada T., Sato T., Harada A. (2010) Induction of liver steatosis and lipid droplet formation in ATF6α-knockout mice burdened with pharmacological endoplasmic reticulum stress. Mol. Biol. Cell 21, 2975–2986 10.1091/mbc.e09-02-0133 20631254 PMC2929991

[37] Promlek T., Ishiwata-Kimata Y., Shido M., Sakuramoto M., Kohno K. and Kimata Y. (2011) Membrane aberrancy and unfolded proteins activate the endoplasmic reticulum stress sensor Ire1 in different ways. Mol. Biol. Cell 22, 3520–3532 10.1091/mbc.e11-04-0295 21775630 PMC3172275

[38] Volmer R., van der Ploeg K. and Ron D. (2013) Membrane lipid saturation activates endoplasmic reticulum unfolded protein response transducers through their transmembrane domains. Proc. Natl. Acad. Sci. U.S.A. 110, 4628–4633 10.1073/pnas.1217611110 23487760 PMC3606975

[39] Halbleib K., Pesek K., Covino R., Hofbauer H.F., Wunnicke D., Hanelt I. (2017) Activation of the unfolded protein response by lipid bilayer stress. Mol. Cell 67, 673e8–684e8 10.1016/j.molcel.2017.06.012 28689662

[40] Thibault G., Ismail N. and Ng D.T. (2011) The unfolded protein response supports cellular robustness as a broad-spectrum compensatory pathway. Proc. Natl. Acad. Sci. U.S.A. 108, 20597–20602 10.1073/pnas.1117184109 22143797 PMC3251055

[41] Lajoie P., Moir R.D., Willis I.M. and Snapp E.L. (2012) Kar2p availability defines distinct forms of endoplasmic reticulum stress in living cells. Mol. Biol. Cell 23, 955–964 10.1091/mbc.e11-12-0995 22219379 PMC3290652

[42] Hou N.S., Gutschmidt A., Choi D.Y., Pather K., Shi X., Watts J.L. (2014) Activation of the endoplasmic reticulum unfolded protein response by lipid disequilibrium without disturbed proteostasis *in vivo*. Proc. Natl. Acad. Sci. U.S.A. 111, E2271–E2280 10.1073/pnas.1318262111 24843123 PMC4050548

[43] Dalton R., Karagoz E., Kahiapo J., Sharma R., Bashkirova L., Lyons D. (2018) Olfactory and vomeronasal receptor feedback employ divergent mechanisms of PERK activation. bioRxiv, 10.1101/239830

[44] Thibault G., Shui G., Kim W., McAlister G.C., Ismail N., Gygi S.P. (2012) The membrane stress response buffers lethal effects of lipid disequilibrium by reprogramming the protein homeostasis network. Mol. Cell 48, 16–27 10.1016/j.molcel.2012.08.016 23000174 PMC3496426

[45] Olzmann J.A. and Kopito R.R. (2011) Lipid droplet formation is dispensable for endoplasmic reticulum-associated degradation. J. Biol. Chem. 286, 27872–27874 10.1074/jbc.C111.266452 21693705 PMC3151032

[46] To M., Peterson C.W., Roberts M.A., Counihan J.L., Wu T.T., Forster M.S. (2017) Lipid disequilibrium disrupts ER proteostasis by impairing ERAD substrate glycan trimming and dislocation. Mol. Biol. Cell 28, 270–284 10.1091/mbc.e16-07-0483 27881664 PMC5231896

[47] Klemm E.J., Spooner E. and Ploegh H.L. (2011) Dual role of ancient ubiquitous protein 1 (AUP1) in lipid droplet accumulation and endoplasmic reticulum (ER) protein quality control. J. Biol. Chem. 286, 37602–37614 10.1074/jbc.M111.284794 21857022 PMC3199505

[48] Ploegh H.L. (2007) A lipid-based model for the creation of an escape hatch from the endoplasmic reticulum. Nature 448, 435–438 10.1038/nature06004 17653186

[49] Hartman I.Z., Liu P., Zehmer J.K., Luby-Phelps K., Jo Y., Anderson R.G. (2010) Sterol-induced dislocation of 3-hydroxy-3-*methylglutaryl* coenzyme A reductase from endoplasmic reticulum membranes into the cytosol through a subcellular compartment resembling lipid droplets. J. Biol. Chem. 285, 19288–19298 10.1074/jbc.M110.134213 20406816 PMC2885207

[50] Shibata M., Kanda M., Tanaka H., Umeda S., Miwa T., Shimizu D. (2017) Overexpression of Derlin 3 is associated with malignant phenotype of breast cancer cells. Oncol. Rep. 38, 1760–1766 10.3892/or.2017.5800 28713959

[51] Wang Q., Mora-Jensen H., Weniger M.A., Perez-Galan P., Wolford C., Hai T. (2009) ERAD inhibitors integrate ER stress with an epigenetic mechanism to activate BH3-only protein NOXA in cancer cells. Proc. Natl. Acad. Sci. U.S.A. 106, 2200–2205 10.1073/pnas.0807611106 19164757 PMC2629785

[52] Shostak A., Meyer-Kovac J. and Oster H. (2013) Circadian regulation of lipid mobilization in white adipose tissues. Diabetes 62, 2195–2203 10.2337/db12-1449 23434933 PMC3712056

[53] Hatori M., Vollmers C., Zarrinpar A., DiTacchio L., Bushong E.A., Gill S. (2012) Time-restricted feeding without reducing caloric intake prevents metabolic diseases in mice fed a high-fat diet. Cell Metab. 15, 848–860 10.1016/j.cmet.2012.04.019 22608008 PMC3491655

[54] Cretenet G., Le Clech M. and Gachon F. (2010) Circadian clock-coordinated 12 Hr period rhythmic activation of the IRE1alpha pathway controls lipid metabolism in mouse liver. Cell Metab. 11, 47–57 10.1016/j.cmet.2009.11.002 20074527

[55] Bu Y., Yoshida A., Chitnis N., Altman B.J., Tameire F., Oran A. (2018) A PERK-miR-211 axis suppresses circadian regulators and protein synthesis to promote cancer cell survival. Nat. Cell Biol. 20, 104–115 10.1038/s41556-017-0006-y 29230015 PMC5741512

[56] Zeng Z.L., Luo H.Y., Yang J., Wu W.J., Chen D.L., Huang P. (2014) Overexpression of the circadian clock gene Bmal1 increases sensitivity to oxaliplatin in colorectal cancer. Clin. Cancer Res. 20, 1042–1052 10.1158/1078-0432.CCR-13-0171 24277452

[57] Lee J., Hong S.W., Kwon H., Park S.E., Rhee E.J., Park C.Y. (2018) Exendin-4 improves ER stress-induced lipid accumulation and regulates lipin-1 signaling in HepG2 cells. Cell Stress Chaperones 23, 629–638 10.1007/s12192-017-0872-z 29934713 PMC6045528

[58] Chen J., Xie J.J., Shi K.S., Gu Y.T., Wu C.C., Xuan J. (2018) Glucagon-like peptide-1 receptor regulates endoplasmic reticulum stress-induced apoptosis and the associated inflammatory response in chondrocytes and the progression of osteoarthritis in rat. Cell Death Dis. 9, 212 10.1038/s41419-017-0217-y 29434185 PMC5833344

[59] He L., Law P.T.Y., Wong C.K., Chan J.C.N. and Chan P.K.S. (2017) Exendin-4 exhibits enhanced anti-tumor effects in diabetic mice. Sci. Rep. 7, 1791 10.1038/s41598-017-01952-5 28496193 PMC5431757

[60] Yin Q.H., Zhang R., Li L., Wang Y.T., Liu J.P., Zhang J. (2016) Exendin-4 ameliorates lipotoxicity-induced glomerular endothelial cell injury by improving ABC transporter A1-mediated cholesterol efflux in diabetic apoE knockout mice. J. Biol. Chem. 291, 26487–26501 10.1074/jbc.M116.730564 27784780 PMC5159509

[61] Gupta N.A., Kolachala V.L., Jiang R., Abramowsky C., Romero R., Fifadara N. (2012) The glucagon-like peptide-1 receptor agonist Exendin 4 has a protective role in ischemic injury of lean and steatotic liver by inhibiting cell death and stimulating lipolysis. Am. J. Pathol. 181, 1693–1701 10.1016/j.ajpath.2012.07.015 22960075 PMC5691335

[62] Lee J., Hong S.W., Chae S.W., Kim D.H., Choi J.H., Bae J.C. (2012) Exendin-4 improves steatohepatitis by increasing Sirt1 expression in high-fat diet-induced obese C57BL/6J mice. PLoS ONE 7, e31394 10.1371/journal.pone.0031394 22363635 PMC3281956

[63] Calvisi D.F., Wang C., Ho C., Ladu S., Lee S.A., Mattu S. (2011) Increased lipogenesis, induced by AKT-mTORC1-RPS6 signaling, promotes development of human hepatocellular carcinoma. Gastroenterology 140, 1071–1083 10.1053/j.gastro.2010.12.006 21147110 PMC3057329

[64] Chajes V., Cambot M., Moreau K., Lenoir G.M. and Joulin V. (2006) Acetyl-CoA carboxylase alpha is essential to breast cancer cell survival. Cancer Res. 66, 5287–5294 10.1158/0008-5472.CAN-05-1489 16707454

[65] Swinnen J.V., Roskams T., Joniau S., Van Poppel H., Oyen R., Baert L. (2002) Overexpression of fatty acid synthase is an early and common event in the development of prostate cancer. Int. J. Cancer 98, 19–22 10.1002/ijc.10127 11857379

[66] Nelson M.E., Lahiri S., Chow J.D., Byrne F.L., Hargett S.R., Breen D.S. (2017) Inhibition of hepatic lipogenesis enhances liver tumorigenesis by increasing antioxidant defence and promoting cell survival. Nat. Commun. 8, 14689 10.1038/ncomms14689 28290443 PMC5424065

[67] Finkel T. and Holbrook N.J. (2000) Oxidants, oxidative stress and the biology of ageing. Nature 408, 239–247 10.1038/35041687 11089981

[68] Lin M.T. and Beal M.F. (2006) Mitochondrial dysfunction and oxidative stress in neurodegenerative diseases. Nature 443, 787–795 10.1038/nature05292 17051205

[69] Halliwell B. (2007) Oxidative stress and cancer: have we moved forward? Biochem. J. 401, 1–11 10.1042/BJ20061131 17150040

[70] Gorrini C., Harris I.S. and Mak T.W. (2013) Modulation of oxidative stress as an anticancer strategy. Nat. Rev. Drug Discov. 12, 931–947 10.1038/nrd4002 24287781

[71] Rhee S.G., Yang K.S., Kang S.W., Woo H.A. and Chang T.S. (2005) Controlled elimination of intracellular H(2)O: regulation of peroxiredoxin, catalase, and glutathione peroxidase via post-translational modification. Antioxid. Redox Signal. 7, 619–626 10.1089/ars.2005.7.619 15890005

[72] D’Autréaux B. and Toledano M.B. (2007) ROS as signalling molecules: mechanisms that generate specificity in ROS homeostasis. Nat. Rev. Mol. Cell Biol. 8, 813–824 10.1038/nrm2256 17848967

[73] Lee S.J., Zhang J., Choi A.M. and Kim H.P. (2013) Mitochondrial dysfunction induces formation of lipid droplets as a generalized response to stress. Oxid. Med. Cell Longev. 2013, 327167 10.1155/2013/327167 24175011 PMC3794647

[74] Liu L., Zhang K., Sandoval H., Yamamoto S., Jaiswal M., Sanz E. (2015) Glial lipid droplets and ROS induced by mitochondrial defects promote neurodegeneration. Cell 160, 177–190 10.1016/j.cell.2014.12.019 25594180 PMC4377295

[75] Zheng P., Xie Z., Yuan Y., Sui W., Wang C., Gao X. (2017) Plin5 alleviates myocardial ischaemia/reperfusion injury by reducing oxidative stress through inhibiting the lipolysis of lipid droplets. Sci. Rep. 7, 42574 10.1038/srep42574 28218306 PMC5316932

[76] Du W., Zhang L., Brett-Morris A., Aguila B., Kerner J., Hoppel C.L. (2017) HIF drives lipid deposition and cancer in ccRCC via repression of fatty acid metabolism. Nat. Commun. 8, 1769 10.1038/s41467-017-01965-8 29176561 PMC5701259

[77] Ott M., Gogvadze V., Orrenius S. and Zhivotovsky B. (2007) Mitochondria, oxidative stress and cell death. Apoptosis 12, 913–922 10.1007/s10495-007-0756-2 17453160

[78] Lee J., Giordano S. and Zhang J. (2012) Autophagy, mitochondria and oxidative stress: cross-talk and redox signalling. Biochem. J. 441, 523–540 10.1042/BJ20111451 22187934 PMC3258656

[79] Paradies G., Petrosillo G., Pistolese M. and Ruggiero F.M. (2002) Reactive oxygen species affect mitochondrial electron transport complex I activity through oxidative cardiolipin damage. Gene 286, 135–141 10.1016/S0378-1119(01)00814-9 11943469

[80] Bailey A.P., Koster G., Guillermier C., Hirst E.M., MacRae J.I., Lechene C.P. (2015) Antioxidant role for lipid droplets in a stem cell niche of Drosophila. Cell 163, 340–353 10.1016/j.cell.2015.09.020 26451484 PMC4601084

[81] Panieri E. and Santoro M.M. (2016) ROS homeostasis and metabolism: a dangerous liason in cancer cells. Cell Death Dis. 7, e2253 10.1038/cddis.2016.105 27277675 PMC5143371

[82] Galadari S., Rahman A., Pallichankandy S. and Thayyullathil F. (2017) Reactive oxygen species and cancer paradox: to promote or to suppress? Free Radic. Biol. Med. 104, 144–164 10.1016/j.freeradbiomed.2017.01.004 28088622

[83] Sabharwal S.S. and Schumacker P.T. (2014) Mitochondrial ROS in cancer: initiators, amplifiers or an Achilles’ heel? Nat. Rev. Cancer 14, 709–721 10.1038/nrc3803 25342630 PMC4657553

[84] Idelchik M., Begley U., Begley T.J. and Melendez J.A. (2017) Mitochondrial ROS control of cancer. Semin. Cancer Biol. 47, 57–66 10.1016/j.semcancer.2017.04.005 28445781 PMC5653465

[85] Zhang X.Y., Hong S.S., Zhang M., Cai Q.Q., Zhang M.X. and Xu C.J. (2018) Proteomic alterations of fibroblasts induced by ovarian cancer cells reveal potential cancer targets. Neoplasma 65, 104–112 10.4149/neo_2018_101 28857608

[86] Patel G.K., Khan M.A., Bhardwaj A., Srivastava S.K., Zubair H., Patton M.C. (2017) Exosomes confer chemoresistance to pancreatic cancer cells by promoting ROS detoxification and miR-155-mediated suppression of key gemcitabine-metabolising enzyme, DCK. Br. J. Cancer 116, 609–619 10.1038/bjc.2017.18 28152544 PMC5344296

[87] Lillig C.H., Lonn M.E., Enoksson M., Fernandes A.P. and Holmgren A. (2004) Short interfering RNA-mediated silencing of glutaredoxin 2 increases the sensitivity of HeLa cells toward doxorubicin and phenylarsine oxide. Proc. Natl. Acad. Sci. U.S.A. 101, 13227–13232 10.1073/pnas.0401896101 15328416 PMC516552

[88] Zhang J., Yao J., Peng S., Li X. and Fang J. (2017) Securinine disturbs redox homeostasis and elicits oxidative stress-mediated apoptosis via targeting thioredoxin reductase. Biochim. Biophys. Acta 1863, 129–138 10.1016/j.bbadis.2016.10.019 27777067

[89] Jarc E., Kump A., Malavasic P., Eichmann T.O., Zimmermann R. and Petan T. (2018) Lipid droplets induced by secreted phospholipase A2 and unsaturated fatty acids protect breast cancer cells from nutrient and lipotoxic stress. Biochim. Biophys. Acta 1863, 247–265 10.1016/j.bbalip.2017.12.006 29229414

[90] Chow J.D., Lawrence R.T., Healy M.E., Dominy J.E., Liao J.A., Breen D.S. (2014) Genetic inhibition of hepatic acetyl-CoA carboxylase activity increases liver fat and alters global protein acetylation. Mol. Metab. 3, 419–431 10.1016/j.molmet.2014.02.004 24944901 PMC4060285

[91] Filosa S., Fico A., Paglialunga F., Balestrieri M., Crooke A., Verde P. (2003) Failure to increase glucose consumption through the pentose-phosphate pathway results in the death of glucose-6-phosphate dehydrogenase gene-deleted mouse embryonic stem cells subjected to oxidative stress. Biochem. J. 370, 935–943 10.1042/bj20021614 12466018 PMC1223222

[92] Juhnke H., Krems B., Kotter P. and Entian K.D. (1996) Mutants that show increased sensitivity to hydrogen peroxide reveal an important role for the pentose phosphate pathway in protection of yeast against oxidative stress. Mol. Gen. Genet. 252, 456–464 10.1007/BF02173011 8879247

[93] Han J., Back S.H., Hur J., Lin Y.H., Gildersleeve R., Shan J. (2013) ER-stress-induced transcriptional regulation increases protein synthesis leading to cell death. Nat. Cell Biol. 15, 481–490 10.1038/ncb2738 23624402 PMC3692270

[94] Puskas L.G., Feher L.Z., Vizler C., Ayaydin F., Raso E., Molnar E. (2010) Polyunsaturated fatty acids synergize with lipid droplet binding thalidomide analogs to induce oxidative stress in cancer cells. Lipids Health Dis. 9, 56 10.1186/1476-511X-9-56 20525221 PMC2902471

[95] Åkerfelt M., Morimoto R.I. and Sistonen L. (2010) Heat shock factors: integrators of cell stress, development and lifespan. Nat. Rev. Mol. Cell Biol. 11, 545–555 10.1038/nrm2938 20628411 PMC3402356

[96] Richter K., Haslbeck M. and Buchner J. (2010) The heat shock response: life on the verge of death. Mol. Cell 40, 253–266 10.1016/j.molcel.2010.10.006 20965420

[97] Gomez-Pastor R., Burchfiel E.T. and Thiele D.J. (2018) Regulation of heat shock transcription factors and their roles in physiology and disease. Nat. Rev. Mol. Cell Biol. 19, 4–19 10.1038/nrm.2017.73 28852220 PMC5794010

[98] Dai C., Whitesell L., Rogers A.B. and Lindquist S. (2007) Heat shock factor 1 is a powerful multifaceted modifier of carcinogenesis. Cell 130, 1005–1018 10.1016/j.cell.2007.07.020 17889646 PMC2586609

[99] Mendillo M.L., Santagata S., Koeva M., Bell G.W., Hu R., Tamimi R.M. (2012) HSF1 drives a transcriptional program distinct from heat shock to support highly malignant human cancers. Cell 150, 549–562 10.1016/j.cell.2012.06.031 22863008 PMC3438889

[100] Scherz-Shouval R., Santagata S., Mendillo M.L., Sholl L.M., Ben-Aharon I., Beck A.H. (2014) The reprogramming of tumor stroma by HSF1 is a potent enabler of malignancy. Cell 158, 564–578 10.1016/j.cell.2014.05.045 25083868 PMC4249939

[101] Vilaboa N., Bore A., Martin-Saavedra F., Bayford M., Winfield N., Firth-Clark S. (2017) New inhibitor targeting human transcription factor HSF1: effects on the heat shock response and tumor cell survival. Nucleic Acids Res. 45, 5797–5817 10.1093/nar/gkx194 28369544 PMC5449623

[102] Yang Z., Zhuang L., Szatmary P., Wen L., Sun H., Lu Y. (2015) Upregulation of heat shock proteins (HSPA12A, HSP90B1, HSPA4, HSPA5 and HSPA6) in tumour tissues is associated with poor outcomes from HBV-related early-stage hepatocellular carcinoma. Int. J. Med. Sci. 12, 256–263 10.7150/ijms.10735 25798051 PMC4366630

[103] Kinzel L., Ernst A., Orth M., Albrecht V., Hennel R., Brix N. (2016) A novel HSP90 inhibitor with reduced hepatotoxicity synergizes with radiotherapy to induce apoptosis, abrogate clonogenic survival, and improve tumor control in models of colorectal cancer. Oncotarget 7, 43199–43219 10.18632/oncotarget.9774 27259245 PMC5190018

[104] McKeon A.M., Egan A., Chandanshive J., McMahon H. and Griffith D.M. (2016) Novel improved synthesis of HSP70 inhibitor, Pifithrin-mu. *In vitro* synergy quantification of Pifithrin-mu combined with Pt drugs in prostate and colorectal cancer cells. Molecules 21, 949 10.3390/molecules21070949 PMC6273252 27455212

[105] Lee Y., Sunada S., Hirakawa H., Fujimori A., Nickoloff J.A. and Okayasu R. (2017) TAS-116, a novel Hsp90 inhibitor, selectively enhances radiosensitivity of human cancer cells to X-rays and carbon ion radiation. Mol. Cancer Ther. 16, 16–24 10.1158/1535-7163.MCT-16-0573 28062703 PMC5221699

[106] Peter M., Glatz A., Gudmann P., Gombos I., Torok Z., Horvath I. (2017) Metabolic crosstalk between membrane and storage lipids facilitates heat stress management in Schizosaccharomyces pombe. PLoS ONE 12, e0173739 10.1371/journal.pone.0173739 28282432 PMC5345867

[107] Armutcu F., Ataymen M., Atmaca H. and Gurel A. (2008) Oxidative stress markers, C-reactive protein and heat shock protein 70 levels in subjects with metabolic syndrome. Clin. Chem. Lab. Med. 46, 785–790 10.1515/CCLM.2008.166 18601599

[108] Jiang H., He J., Pu S., Tang C. and Xu G. (2007) Heat shock protein 70 is translocated to lipid droplets in rat adipocytes upon heat stimulation. Biochim. Biophys. Acta 1771, 66–74 10.1016/j.bbalip.2006.10.004 17175194

[109] Balogh G., Maulucci G., Gombos I., Horvath I., Torok Z., Peter M. (2011) Heat stress causes spatially-distinct membrane re-modelling in K562 leukemia cells. PLoS ONE 6, e21182 10.1371/journal.pone.0021182 21698159 PMC3116874

[110] van Ginkel G., van Langen H. and Levine Y.K. (1989) The membrane fluidity concept revisited by polarized fluorescence spectroscopy on different model membranes containing unsaturated lipids and sterols. Biochimie 71, 23–32 10.1016/0300-9084(89)90127-2 2497794

[111] Kahya N., Scherfeld D., Bacia K., Poolman B. and Schwille P. (2003) Probing lipid mobility of raft-exhibiting model membranes by fluorescence correlation spectroscopy. J. Biol. Chem. 278, 28109–28115 10.1074/jbc.M302969200 12736276

[112] Crane J.M. and Tamm L.K. (2004) Role of cholesterol in the formation and nature of lipid rafts in planar and spherical model membranes. Biophys. J. 86, 2965–2979 10.1016/S0006-3495(04)74347-7 15111412 PMC1304164

[113] Subczynski W.K., Pasenkiewicz-Gierula M., Widomska J., Mainali L. and Raguz M. (2017) High cholesterol/low cholesterol: effects in biological membranes review. Cell Biochem. Biophys. 1–17 28417231 10.1007/s12013-017-0792-7 PMC5645210

[114] Torrano V., Valcarcel-Jimenez L., Cortazar A.R., Liu X., Urosevic J., Castillo-Martin M. (2016) The metabolic co-regulator PGC1alpha suppresses prostate cancer metastasis. Nat. Cell Biol. 18, 645–656 10.1038/ncb3357 27214280 PMC4884178

[115] Minsky N. and Roeder R.G. (2015) Direct link between metabolic regulation and the heat-shock response through the transcriptional regulator PGC-1alpha. Proc. Natl. Acad. Sci. U.S.A. 112, E5669–E5678 10.1073/pnas.1516219112 26438876 PMC4620912

[116] Gallardo-Montejano V.I., Saxena G., Kusminski C.M., Yang C., McAfee J.L., Hahner L. (2016) Nuclear Perilipin 5 integrates lipid droplet lipolysis with PGC-1alpha/SIRT1-dependent transcriptional regulation of mitochondrial function. Nat. Commun. 7, 12723 10.1038/ncomms12723 27554864 PMC4999519

[117] Ciocca D.R., Fuqua S.A., Lock-Lim S., Toft D.O., Welch W.J. and McGuire W.L. (1992) Response of human breast cancer cells to heat shock and chemotherapeutic drugs. Cancer Res. 52, 3648–3654 1617638

[118] Donaldson S.S., Gordon L.F. and Hahn G.M. (1978) Protective effect of hyperthermia against the cytotoxicity of actinomycin D on Chinese hamster cells. Cancer Treat. Rep. 62, 1489–1495 709551

[119] Zhang J., Fan N. and Peng Y. (2018) Heat shock protein 70 promotes lipogenesis in HepG2 cells. Lipids Health Dis. 17, 73 10.1186/s12944-018-0722-8 29631603 PMC5891916

[120] Yu W., Bozza P.T., Tzizik D.M., Gray J.P., Cassara J., Dvorak A.M. (1998) Co-compartmentalization of MAP kinases and cytosolic phospholipase A2 at cytoplasmic arachidonate-rich lipid bodies. Am. J. Pathol. 152, 759–769 9502418 PMC1858398

[121] Jurivich D.A., Sistonen L., Sarge K.D. and Morimoto R.I. (1994) Arachidonate is a potent modulator of human heat shock gene transcription. Proc. Natl. Acad. Sci. U.S.A. 91, 2280–2284 10.1073/pnas.91.6.2280 8134388 PMC43354

[122] Balogh G., Peter M., Liebisch G., Horvath I., Torok Z., Nagy E. (2010) Lipidomics reveals membrane lipid remodelling and release of potential lipid mediators during early stress responses in a murine melanoma cell line. Biochim. Biophys. Acta 1801, 1036–1047 10.1016/j.bbalip.2010.04.011 20430110

[123] Moses M.A., Kim Y.S., Rivera-Marquez G.M., Oshima N., Watson M.J., Beebe K.E. (2018) Targeting the Hsp40/Hsp70 chaperone axis as a novel strategy to treat castration-resistant prostate cancer. Cancer Res. 78, 4022–4035 10.1158/0008-5472.CAN-17-3728 29764864 PMC6050126

[124] Li Z., Qu M., Sun Y., Wan H., Chai F., Liu L. (2018) Blockage of cytosolic phospholipase A2 alpha sensitizes aggressive breast cancer to doxorubicin through suppressing ERK and mTOR kinases. Biochem. Biophys. Res. Commun. 496, 153–158 10.1016/j.bbrc.2018.01.016 29307829

[125] Hanahan D. and Weinberg R.A. (2011) Hallmarks of cancer: the next generation. Cell 144, 646–674 10.1016/j.cell.2011.02.013 21376230

[126] Abramczyk H., Surmacki J., Kopec M., Olejnik A.K., Lubecka-Pietruszewska K. and Fabianowska-Majewska K. (2015) The role of lipid droplets and adipocytes in cancer. Raman imaging of cell cultures: MCF10A, MCF7, and MDA-MB-231 compared to adipocytes in cancerous human breast tissue. Analyst 140, 2224–2235 10.1039/C4AN01875C 25730442

[127] Nieva C., Marro M., Santana-Codina N., Rao S., Petrov D. and Sierra A. (2012) The lipid phenotype of breast cancer cells characterized by Raman microspectroscopy: towards a stratification of malignancy. PLoS ONE 7, e46456 10.1371/journal.pone.0046456 23082122 PMC3474759

[128] Ackerman D. and Simon M.C. (2014) Hypoxia, lipids, and cancer: surviving the harsh tumor microenvironment. Trends Cell Biol. 24, 472–478 10.1016/j.tcb.2014.06.001 24985940 PMC4112153

[129] Baenke F., Peck B., Miess H. and Schulze A. (2013) Hooked on fat: the role of lipid synthesis in cancer metabolism and tumour development. Dis. Model Mech. 6, 1353–1363 10.1242/dmm.011338 24203995 PMC3820259

[130] Cruz P.M., Mo H., McConathy W.J., Sabnis N. and Lacko A.G. (2013) The role of cholesterol metabolism and cholesterol transport in carcinogenesis: a review of scientific findings, relevant to future cancer therapeutics. Front. Pharmacol. 4, 119 10.3389/fphar.2013.00119 24093019 PMC3782849

[131] Antalis C.J., Uchida A., Buhman K.K. and Siddiqui R.A. (2011) Migration of MDA-MB-231 breast cancer cells depends on the availability of exogenous lipids and cholesterol esterification. Clin. Exp. Metastasis 28, 733–741 10.1007/s10585-011-9405-9 21744083

[132] Mitra R., Chao O., Urasaki Y., Goodman O.B. and Le T.T. (2012) Detection of lipid-rich prostate circulating tumour cells with coherent anti-Stokes Raman scattering microscopy. BMC Cancer 12, 540 10.1186/1471-2407-12-540 23171028 PMC3519750

[133] Yue S., Li J., Lee S.Y., Lee H.J., Shao T., Song B. (2014) Cholesteryl ester accumulation induced by PTEN loss and PI3K/AKT activation underlies human prostate cancer aggressiveness. Cell Metab. 19, 393–406 10.1016/j.cmet.2014.01.019 24606897 PMC3969850

[134] Krause B.R. and Hartman A.D. (1984) Adipose tissue and cholesterol metabolism. J. Lipid Res. 25, 97–110 6368715

[135] George R. and Ramasarma T. (1977) Nature of the stimulation of biogenesis of cholesterol in the liver by noradrenaline. Biochem. J. 162, 493–499 10.1042/bj1620493 68775 PMC1164632

[136] Mitsche M.A., McDonald J.G., Hobbs H.H. and Cohen J.C. (2015) Flux analysis of cholesterol biosynthesis *in vivo* reveals multiple tissue and cell-type specific pathways. Elife 4, e07999 10.7554/eLife.07999 26114596 PMC4501332

[137] Lorick K.L., Jensen J.P., Fang S., Ong A.M., Hatakeyama S. and Weissman A.M. (1999) RING fingers mediate ubiquitin-conjugating enzyme (E2)-dependent ubiquitination. Proc. Natl. Acad. Sci. U.S.A. 96, 11364–11369 10.1073/pnas.96.20.11364 10500182 PMC18039

[138] Gemmill R.M., West J.D., Boldog F., Tanaka N., Robinson L.J., Smith D.I. (1998) The hereditary renal cell carcinoma 3;8 translocation fuses FHIT to a patched-related gene, TRC8. Proc. Natl. Acad. Sci. U.S.A. 95, 9572–9577 10.1073/pnas.95.16.9572 9689122 PMC21380

[139] Gimelli S., Beri S., Drabkin H.A., Gambini C., Gregorio A., Fiorio P. (2009) The tumor suppressor gene TRC8/RNF139 is disrupted by a constitutional balanced translocation t(8;22)(q24.13;q11.21) in a young girl with dysgerminoma. Mol. Cancer 8, 52 10.1186/1476-4598-8-52 19642973 PMC2727492

[140] Poland K.S., Azim M., Folsom M., Goldfarb R., Naeem R., Korch C. (2007) A constitutional balanced t(3;8)(p14;q24.1) translocation results in disruption of the TRC8 gene and predisposition to clear cell renal cell carcinoma. Genes Chromosomes Cancer 46, 805–812 10.1002/gcc.20466 17539022

[141] Gang X., Yang Y., Zhong J., Jiang K., Pan Y., Karnes R.J. (2016) P300 acetyltransferase regulates fatty acid synthase expression, lipid metabolism and prostate cancer growth. Oncotarget 7, 15135–15149 10.18632/oncotarget.7715 26934656 PMC4924775

[142] Huang W.C., Li X., Liu J., Lin J. and Chung L.W. (2012) Activation of androgen receptor, lipogenesis, and oxidative stress converged by SREBP-1 is responsible for regulating growth and progression of prostate cancer cells. Mol. Cancer Res. 10, 133–142 10.1158/1541-7786.MCR-11-0206 22064655 PMC3262123

[143] Potcoava M.C., Futia G.L., Aughenbaugh J., Schlaepfer I.R. and Gibson E.A. (2014) Raman and coherent anti-Stokes Raman scattering microscopy studies of changes in lipid content and composition in hormone-treated breast and prostate cancer cells. J. Biomed. Opt. 19, 111605 10.1117/1.JBO.19.11.111605 24933682 PMC4059341

[144] Griffin J.L. and Shockcor J.P. (2004) Metabolic profiles of cancer cells. Nat. Rev. Cancer 4, 551 10.1038/nrc1390 15229480

[145] Liberti M.V. and Locasale J.W. (2016) The Warburg effect: how does it benefit cancer cells? Trends Biochem. Sci. 41, 211–218 10.1016/j.tibs.2015.12.001 26778478 PMC4783224

[146] Cairns R.A., Harris I.S. and Mak T.W. (2011) Regulation of cancer cell metabolism. Nat. Rev. Cancer 11, 85–95 10.1038/nrc2981 21258394

[147] Beloribi-Djefaflia S., Vasseur S. and Guillaumond F. (2016) Lipid metabolic reprogramming in cancer cells. Oncogenesis 5, e189 10.1038/oncsis.2015.49 26807644 PMC4728678

[148] Menendez J.A. and Lupu R. (2007) Fatty acid synthase and the lipogenic phenotype in cancer pathogenesis. Nat. Rev. Cancer 7, 763–777 10.1038/nrc2222 17882277

[149] Zaidi N., Swinnen J.V. and Smans K. (2012) ATP-citrate lyase: a key player in cancer metabolism. Cancer Res. 72, 3709–3714 10.1158/0008-5472.CAN-11-4112 22787121

[150] Kuhajda F.P. (2006) Fatty acid synthase and cancer: new application of an old pathway. Cancer Res. 66, 5977–5980 10.1158/0008-5472.CAN-05-4673 16778164

[151] Bozza P.T. and Viola J.P. (2010) Lipid droplets in inflammation and cancer. Prostaglandins Leukot. Essent. Fatty Acids 82, 243–250 10.1016/j.plefa.2010.02.005 20206487

[152] Barba I., Cabañas M.E. and Arús C. (1999) The relationship between nuclear magnetic resonance-visible lipids, lipid droplets, and cell proliferation in cultured C6 cells. Cancer Res. 59, 1861–1868 10213493

[153] Koizume S. and Miyagi Y. (2016) Lipid droplets: a key cellular organelle associated with cancer cell survival under normoxia and hypoxia. Int. J. Mol. Sci. 17, 10.3390/ijms17091430 27589734 PMC5037709

[154] Nieman K.M., Kenny H.A., Penicka C.V., Ladanyi A., Buell-Gutbrod R., Zillhardt M.R. (2011) Adipocytes promote ovarian cancer metastasis and provide energy for rapid tumor growth. Nat. Med. 17, 1498–1503 10.1038/nm.2492 22037646 PMC4157349

[155] Yan F., Shen N., Pang J.X., Zhang Y.W., Rao E.Y., Bode A.M. (2017) Fatty acid-binding protein FABP4 mechanistically links obesity with aggressive AML by enhancing aberrant DNA methylation in AML cells. Leukemia 31, 1434–1442 10.1038/leu.2016.349 27885273 PMC5457366

[156] Gazi E., Gardner P., Lockyer N.P., Hart C.A., Brown M.D. and Clarke N.W. (2007) Direct evidence of lipid translocation between adipocytes and prostate cancer cells with imaging FTIR microspectroscopy. J. Lipid Res. 48, 1846–1856 10.1194/jlr.M700131-JLR200 17496269

[157] Balaban S., Shearer R.F., Lee L.S., van Geldermalsen M., Schreuder M., Shtein H.C. (2017) Adipocyte lipolysis links obesity to breast cancer growth: adipocyte-derived fatty acids drive breast cancer cell proliferation and migration. Cancer Metab. 5, 1 10.1186/s40170-016-0163-7 28101337 PMC5237166

[158] Herber D.L., Cao W., Nefedova Y., Novitskiy S.V., Nagaraj S., Tyurin V.A. (2010) Lipid accumulation and dendritic cell dysfunction in cancer. Nat. Med. 16, 880–886 10.1038/nm.2172 20622859 PMC2917488

[159] Cubillos-Ruiz J.R., Silberman P.C., Rutkowski M.R., Chopra S., Perales-Puchalt A., Song M. (2015) ER stress sensor XBP1 controls anti-tumor immunity by disrupting dendritic cell homeostasis. Cell 161, 1527–1538 10.1016/j.cell.2015.05.025 26073941 PMC4580135

[160] Nagaraj S., Pisarev V., Kinarsky L., Sherman S., Muro-Cacho C., Altieri D.C. (2007) Dendritic cell-based full-length survivin vaccine in treatment of experimental tumors. J. Immunother. 30, 169–179 10.1097/01.cji.0000211329.83890.ba 17471164

[161] Cermelli S., Guo Y., Gross S.P. and Welte M.A. (2006) The lipid-droplet proteome reveals that droplets are a protein-storage depot. Curr. Biol. 16, 1783–1795 10.1016/j.cub.2006.07.062 16979555

[162] Wang D. and Dubois R.N. (2010) Eicosanoids and cancer. Nat. Rev. Cancer 10, 181–193 10.1038/nrc2809 20168319 PMC2898136

[163] Accioly M.T., Pacheco P., Maya-Monteiro C.M., Carrossini N., Robbs B.K., Oliveira S.S. (2008) Lipid bodies are reservoirs of cyclooxygenase-2 and sites of prostaglandin-E2 synthesis in colon cancer cells. Cancer Res. 68, 1732–1740 10.1158/0008-5472.CAN-07-1999 18339853

[164] Dvorak A.M., Weller P.F., Harvey S., Morgan E.S. and Dvorak H.F. (1993) Ultrastructural localization of prostaglandin endoperoxide synthase (cyclooxygenase) to isolated, purified fractions of guinea pig peritoneal macrophage and line 10 hepatocarcinoma cell lipid bodies. Int. Arch. Allergy Immunol. 101, 136–142 10.1159/000236511 8508051

[165] McLemore T.L., Hubbard W.C., Litterst C.L., Liu M.C., Miller S., McMahon N.A. (1988) Profiles of prostaglandin biosynthesis in normal lung and tumor tissue from lung cancer patients. Cancer Res. 48, 3140–3147 3130187

[166] Wang D. and DuBois R.N. (2004) Cyclooxygenase-2: a potential target in breast cancer. Semin Oncol. 31, 64–73 10.1053/j.seminoncol.2004.01.008 15052544

[167] Greenhough A., Smartt H.J., Moore A.E., Roberts H.R., Williams A.C., Paraskeva C. (2009) The COX-2/PGE 2 pathway: key roles in the hallmarks of cancer and adaptation to the tumour microenvironment. Carcinogenesis 30, 377–386 10.1093/carcin/bgp014 19136477

[168] Prima V., Kaliberova L.N., Kaliberov S., Curiel D.T. and Kusmartsev S. (2017) COX2/mPGES1/PGE2 pathway regulates PD-L1 expression in tumor-associated macrophages and myeloid-derived suppressor cells. Proc. Natl. Acad. Sci. U.S.A., 114 201612920 10.1073/pnas.1612920114 PMC5293015 28096371

[169] Bottcher J.P., Bonavita E., Chakravarty P., Blees H., Cabeza-Cabrerizo M., Sammicheli S. (2018) NK Cells stimulate recruitment of cDC1 into the tumor Microenvironment promoting cancer immune control. Cell 172, 1022e14–1037e14 10.1016/j.cell.2018.01.004 29429633 PMC5847168

[170] Mao Y., Sarhan D., Steven A., Seliger B., Kiessling R. and Lundqvist A. (2014) Inhibition of tumor-derived prostaglandin-e2 blocks the induction of myeloid-derived suppressor cells and recovers natural killer cell activity. Clin. Cancer Res. 20, 4096–4106 10.1158/1078-0432.CCR-14-0635 24907113

[171] Obermajer N., Muthuswamy R., Odunsi K., Edwards R.P. and Kalinski P. (2011) PGE(2)-induced CXCL12 production and CXCR4 expression controls the accumulation of human MDSCs in ovarian cancer environment. Cancer Res. 71, 7463–7470 10.1158/0008-5472.CAN-11-2449 22025564 PMC4993027

[172] Li J., Ren S., Piao H.L., Wang F., Yin P., Xu C. (2016) Integration of lipidomics and transcriptomics unravels aberrant lipid metabolism and defines cholesteryl oleate as potential biomarker of prostate cancer. Sci. Rep. 6, 20984 10.1038/srep20984 26865432 PMC4750101

[173] Casanovas A., Sprenger R.R., Tarasov K., Ruckerbauer D.E., Hannibal-Bach H.K., Zanghellini J. (2015) Quantitative analysis of proteome and lipidome dynamics reveals functional regulation of global lipid metabolism. Chem. Biol. 22, 412–425 10.1016/j.chembiol.2015.02.007 25794437

[174] Koike K., Tsutsumi T., Miyoshi H., Shinzawa S., Shintani Y., Fujie H. (2008) Molecular basis for the synergy between alcohol and hepatitis C virus in hepatocarcinogenesis. J. Gastroenterol. Hepatol. 23, S87–S91 10.1111/j.1440-1746.2007.05292.x 18336672

[175] Moriya K., Fujie H., Shintani Y., Yotsuyanagi H., Tsutsumi T., Ishibashi K. (1998) The core protein of hepatitis C virus induces hepatocellular carcinoma in transgenic mice. Nat. Med. 4, 1065–1067 10.1038/2053 9734402

[176] Miyanari Y., Atsuzawa K., Usuda N., Watashi K., Hishiki T., Zayas M. (2007) The lipid droplet is an important organelle for hepatitis C virus production. Nat. Cell Biol. 9, 1089 10.1038/ncb1631 17721513

[177] Akil A., Peng J., Omrane M., Gondeau C., Desterke C., Marin M. (2016) Septin 9 induces lipid droplets growth by a phosphatidylinositol-5-phosphate and microtubule-dependent mechanism hijacked by HCV. Nat. Commun. 7, 12203 10.1038/ncomms12203 27417143 PMC4947189

[178] Straub B.K., Herpel E., Singer S., Zimbelmann R., Breuhahn K., Macher-Goeppinger S. (2010) Lipid droplet-associated PAT-proteins show frequent and differential expression in neoplastic steatogenesis. Mod. Pathol. 23, 480 10.1038/modpathol.2009.191 20081801

[179] Herker E., Harris C., Hernandez C., Carpentier A., Kaehlcke K., Rosenberg A.R. (2010) Efficient hepatitis C virus particle formation requires diacylglycerol acyltransferase-1. Nat. Med. 16, 1295 10.1038/nm.2238 20935628 PMC3431199

[180] Carvalho F.A., Carneiro F.A., Martins I.C., Assuncao-Miranda I., Faustino A.F., Pereira R.M. (2012) Dengue virus capsid protein binding to hepatic lipid droplets (LD) is potassium ion dependent and is mediated by LD surface proteins. J. Virol. 86, 2096–2108 10.1128/JVI.06796-11 22130547 PMC3302401

[181] Heaton N.S. and Randall G. (2011) Dengue virus and autophagy. Viruses 3, 1332–1341 10.3390/v3081332 21994782 PMC3185800

[182] Heaton N.S. and Randall G. (2010) Dengue virus-induced autophagy regulates lipid metabolism. Cell Host Microbe 8, 422–432 10.1016/j.chom.2010.10.006 21075353 PMC3026642

[183] Samsa M.M., Mondotte J.A., Iglesias N.G., Assuncao-Miranda I., Barbosa-Lima G., Da Poian A.T. (2009) Dengue virus capsid protein usurps lipid droplets for viral particle formation. PLoS Pathog. 5, e1000632 10.1371/journal.ppat.1000632 19851456 PMC2760139

[184] Li Z., Thiel K., Thul P.J., Beller M., Kuhnlein R.P. and Welte M.A. (2012) Lipid droplets control the maternal histone supply of Drosophila embryos. Curr. Biol. 22, 2104–2113 10.1016/j.cub.2012.09.018 23084995 PMC3513403

[185] Cheung W., Gill M., Esposito A., Kaminski C.F., Courousse N., Chwetzoff S. (2010) Rotaviruses associate with cellular lipid droplet components to replicate in viroplasms, and compounds disrupting or blocking lipid droplets inhibit viroplasm formation and viral replication. J. Virol. 84, 6782–6798 10.1128/JVI.01757-09 20335253 PMC2903253

[186] Na T.Y., Shin Y.K., Roh K.J., Kang S.A., Hong I., Oh S.J. (2009) Liver X receptor mediates hepatitis B virus X protein-induced lipogenesis in hepatitis B virus-associated hepatocellular carcinoma. Hepatology 49, 1122–1131 10.1002/hep.22740 19105208

[187] Kim K., Kim K.H., Kim H.H. and Cheong J. (2008) Hepatitis B virus X protein induces lipogenic transcription factor SREBP1 and fatty acid synthase through the activation of nuclear receptor LXRalpha. Biochem. J. 416, 219–230 10.1042/BJ20081336 18782084

[188] Kim K.H., Shin H.J., Kim K., Choi H.M., Rhee S.H., Moon H.B. (2007) Hepatitis B virus X protein induces hepatic steatosis via transcriptional activation of SREBP1 and PPARgamma. Gastroenterology 132, 1955–1967 10.1053/j.gastro.2007.03.039 17484888

[189] Petruzzelli M. and Wagner E.F. (2016) Mechanisms of metabolic dysfunction in cancer-associated cachexia. Genes Dev. 30, 489–501 10.1101/gad.276733.115 26944676 PMC4782044

[190] Tisdale M.J. (2002) Cachexia in cancer patients. Nat. Rev. Cancer 2, 862–871 10.1038/nrc927 12415256

[191] Garcia J.M., Scherer T., Chen J.A., Guillory B., Nassif A., Papusha V. (2013) Inhibition of cisplatin-induced lipid catabolism and weight loss by ghrelin in male mice. Endocrinology 154, 3118–3129 10.1210/en.2013-1179 23832960 PMC3749475

[192] Barreto R., Waning D.L., Gao H., Liu Y., Zimmers T.A. and Bonetto A. (2016) Chemotherapy-related cachexia is associated with mitochondrial depletion and the activation of ERK1/2 and p38 MAPKs. Oncotarget 7, 43442–43460 10.18632/oncotarget.9779 27259276 PMC5190036

[193] Silvério R., Lira F.S., Oyama L.M., do Nascimento C.M.O., Otoch J.P., Alcântara P.S. (2017) Lipases and lipid droplet-associated protein expression in subcutaneous white adipose tissue of cachectic patients with cancer. Lipids Health Dis. 16, 159 10.1186/s12944-017-0547-x 28830524 PMC5568087

[194] Das S.K., Eder S., Schauer S., Diwoky C., Temmel H., Guertl B. (2011) Adipose triglyceride lipase contributes to cancer-associated cachexia. Science 333, 233–238 10.1126/science.1198973 21680814

[195] Saely C.H., Geiger K. and Drexel H. (2012) Brown versus white adipose tissue: a mini-review. Gerontology 58, 15–23 10.1159/000321319 21135534

[196] Das S.K. and Hoefler G. (2013) The role of triglyceride lipases in cancer associated cachexia. Trends Mol. Med. 19, 292–301 10.1016/j.molmed.2013.02.006 23499576 PMC3655383

[197] Bing C., Russell S., Becket E., Pope M., Tisdale M., Trayhurn P. (2006) Adipose atrophy in cancer cachexia: morphologic and molecular analysis of adipose tissue in tumour-bearing mice. Br. J. Cancer 95, 1028–1037 10.1038/sj.bjc.6603360 17047651 PMC2360696

[198] Vaitkus J.A. and Celi F.S. (2017) The role of adipose tissue in cancer-associated cachexia. Exp. Biol. Med. 242, 473–481 10.1177/1535370216683282 PMC5367652 27932592

[199] Stephens N.A., Skipworth R.J., MacDonald A.J., Greig C.A., Ross J.A. and Fearon K.C. (2011) Intramyocellular lipid droplets increase with progression of cachexia in cancer patients. J. Cachexia Sarcopenia Muscle 2, 111–117 10.1007/s13539-011-0030-x 21766057 PMC3117997

[200] Boren J. and Brindle K.M. (2012) Apoptosis-induced mitochondrial dysfunction causes cytoplasmic lipid droplet formation. Cell Death Differ. 19, 1561–1570 10.1038/cdd.2012.34 22460322 PMC3422477

[201] Zirath H., Frenzel A., Oliynyk G., Segerstrom L., Westermark U.K., Larsson K. (2013) MYC inhibition induces metabolic changes leading to accumulation of lipid droplets in tumor cells. Proc. Natl. Acad. Sci. U.S.A. 110, 10258–10263 10.1073/pnas.1222404110 23733953 PMC3690852

[202] Mehdizadeh A., Bonyadi M., Darabi M., Rahbarghazi R., Montazersaheb S., Velaei K. (2017) Common chemotherapeutic agents modulate fatty acid distribution in human hepatocellular carcinoma and colorectal cancer cells. Bioimpacts 7, 31–39 10.15171/bi.2017.05 28546951 PMC5439387

[203] Magtanong L., Ko P.J. and Dixon S.J. (2016) Emerging roles for lipids in non-apoptotic cell death. Cell Death Differ. 23, 1099–1109 10.1038/cdd.2016.25 26967968 PMC5399169

[204] Agmon E. and Stockwell B.R. (2017) Lipid homeostasis and regulated cell death. Curr. Opin. Chem. Biol. 39, 83–89 10.1016/j.cbpa.2017.06.002 28645028 PMC5581689

[205] Dixon S.J., Winter G.E., Musavi L.S., Lee E.D., Snijder B., Rebsamen M. (2015) Human haploid cell genetics reveals roles for lipid metabolism genes in nonapoptotic cell death. ACS Chem. Biol. 10, 1604–1609 10.1021/acschembio.5b00245 25965523 PMC4509420

[206] Pizer E.S., Thupari J., Han W.F., Pinn M.L., Chrest F.J., Frehywot G.L. (2000) Malonyl-coenzyme-A is a potential mediator of cytotoxicity induced by fatty-acid synthase inhibition in human breast cancer cells and xenografts. Cancer Res. 60, 213–218 10667561

[207] Ewald J.A., Desotelle J.A., Wilding G. and Jarrard D.F. (2010) Therapy-induced senescence in cancer. J. Natl. Cancer Inst. 102, 1536–1546 10.1093/jnci/djq364 20858887 PMC2957429

[208] Wang H., Peng G., Bai J., He B., Huang K., Hu X. (2017) Cytomegalovirus infection and relative risk of cardiovascular disease (ischemic heart disease, stroke, and cardiovascular death): a meta-analysis of prospective studies up to 2016. J. Am. Heart Assoc. 6, e005025, 10.1161/JAHA.116.005025 28684641 PMC5586265

[209] Flor A.C., Wolfgeher D., Wu D. and Kron S.J. (2017) A signature of enhanced lipid metabolism, lipid peroxidation and aldehyde stress in therapy-induced senescence. Cell Death Discov. 3, 17075 10.1038/cddiscovery.2017.75 29090099 PMC5661608

[210] Vegiopoulos A., Rohm M. and Herzig S. (2017) Adipose tissue: between the extremes. EMBO J. 36, 1999–2017 10.15252/embj.201696206 28623240 PMC5509999

[211] Havas K.M., Milchevskaya V., Radic K., Alladin A., Kafkia E., Garcia M. (2017) Metabolic shifts in residual breast cancer drive tumor recurrence. J. Clin. Invest. 127, 2091–2105 10.1172/JCI89914 28504653 PMC5451224

[212] Viale A., Pettazzoni P., Lyssiotis C.A., Ying H., Sanchez N., Marchesini M. (2014) Oncogene ablation-resistant pancreatic cancer cells depend on mitochondrial function. Nature 514, 628–632 10.1038/nature13611 25119024 PMC4376130

[213] Cotte A.K., Aires V., Fredon M., Limagne E., Derangere V., Thibaudin M. (2018) Lysophosphatidylcholine acyltransferase 2-mediated lipid droplet production supports colorectal cancer chemoresistance. Nat. Commun. 9, 322 10.1038/s41467-017-02732-5 29358673 PMC5778070

[214] Nakagawa T., Zhu H., Morishima N., Li E., Xu J., Yankner B.A. (2000) Caspase-12 mediates endoplasmic-reticulum-specific apoptosis and cytotoxicity by amyloid-beta. Nature 403, 98–103 10.1038/47513 10638761

[215] Kaini R.R., Sillerud L.O., Zhaorigetu S. and Hu C.A.A. (2012) Autophagy regulates lipolysis and cell survival through lipid droplet degradation in androgen‐sensitive prostate cancer cells. Prostate 72, 1412–1422 10.1002/pros.22489 22294520 PMC3418419

[216] Peck B. and Schulze A. (2014) Cholesteryl esters: fueling the fury of prostate cancer. Cell Metab. 19, 350–352 10.1016/j.cmet.2014.02.012 24606894

[217] Currie E., Schulze A., Zechner R., Walther T.C. and Farese R.V. Jr (2013) Cellular fatty acid metabolism and cancer. Cell Metab. 18, 153–161 10.1016/j.cmet.2013.05.017 23791484 PMC3742569

[218] Hatzivassiliou G., Zhao F., Bauer D.E., Andreadis C., Shaw A.N., Dhanak D. (2005) ATP citrate lyase inhibition can suppress tumor cell growth. Cancer Cell 8, 311–321 10.1016/j.ccr.2005.09.008 16226706

[219] Flavin R., Peluso S., Nguyen P.L. and Loda M. (2010) Fatty acid synthase as a potential therapeutic target in cancer. Future Oncol. 6, 551–562 10.2217/fon.10.11 20373869 PMC3197858

[220] Li S., Qiu L., Wu B., Shen H., Zhu J., Zhou L. (2013) TOFA suppresses ovarian cancer cell growth *in vitro* and *in vivo*. Mol. Med. Rep. 8, 373–378 10.3892/mmr.2013.1505 23732836

[221] Fritz V., Benfodda Z., Rodier G., Henriquet C., Iborra F., Avances C. (2010) Abrogation of *de novo* lipogenesis by stearoyl-CoA desaturase 1 inhibition interferes with oncogenic signaling and blocks prostate cancer progression in mice. Mol. Cancer Ther. 9, 1740–1754 10.1158/1535-7163.MCT-09-1064 20530718 PMC3315476

[222] Schlaepfer I.R., Rider L., Rodrigues L.U., Gijon M.A., Pac C.T., Romero L. (2014) Lipid catabolism via CPT1 as a therapeutic target for prostate cancer. Mol. Cancer Ther. 13, 2361–2371 10.1158/1535-7163.MCT-14-0183 25122071 PMC4185227

[223] Samudio I., Harmancey R., Fiegl M., Kantarjian H., Konopleva M., Korchin B. (2010) Pharmacologic inhibition of fatty acid oxidation sensitizes human leukemia cells to apoptosis induction. J. Clin. Invest. 120, 142–156 10.1172/JCI38942 20038799 PMC2799198

[224] Ricciardi M.R., Mirabilii S., Allegretti M., Licchetta R., Calarco A., Torrisi M.R. (2015) Targeting the leukemia cell metabolism by the CPT1a inhibition: functional preclinical effects in leukemias. Blood 126, 1925–1929 10.1182/blood-2014-12-617498 26276667

[225] Williams K.J., Argus J.P., Zhu Y., Wilks M.Q., Marbois B.N., York A.G. (2013) An essential requirement for the SCAP/SREBP signaling axis to protect cancer cells from lipotoxicity. Cancer Res. 73, 2850–2862 10.1158/0008-5472.CAN-13-0382-T 23440422 PMC3919498

[226] Listenberger L.L., Han X., Lewis S.E., Cases S., Farese R.V. Jr, Ory D.S. (2003) Triglyceride accumulation protects against fatty acid-induced lipotoxicity. Proc. Natl. Acad. Sci. U.S.A. 100, 3077–3082 10.1073/pnas.0630588100 12629214 PMC152249

